# Gene Expression Studies to Identify Significant Genes in AR, MTOR, MAPK Pathways and their Overlapping Regulatory Role in Prostate Cancer

**DOI:** 10.1515/jib-2018-0080

**Published:** 2019-05-28

**Authors:** Nimisha Asati, Abhinav Mishra, Ankita Shukla, Tiratha Raj Singh

**Affiliations:** Department of Biotechnology and Bioinformatics, Jaypee University of Information Technology (JUIT), Waknaghat, Solan, HP, India; ^a^Joint First Authors.

**Keywords:** Prostate cancer, Meta-analysis, Microarray, AR, MAPK, mTOR, Pathways

## Abstract

Gene expression studies revealed a large degree of variability in gene expression patterns particularly in tissues even in genetically identical individuals. It helps to reveal the components majorly fluctuating during the disease condition. With the advent of gene expression studies many microarray studies have been conducted in prostate cancer, but the results have varied across different studies. To better understand the genetic and biological regulatory mechanisms of prostate cancer, we conducted a meta-analysis of three major pathways i.e. androgen receptor (AR), mechanistic target of rapamycin (mTOR) and Mitogen-Activated Protein Kinase (MAPK) on prostate cancer. Meta-analysis has been performed for the gene expression data for the human species that are exposed to prostate cancer. Twelve datasets comprising AR, mTOR, and MAPK pathways were taken for analysis, out of which thirteen potential biomarkers were identified through meta-analysis. These findings were compiled based upon the quantitative data analysis by using different tools. Also, various interconnections were found amongst the pathways in study. Our study suggests that the microarray analysis of the gene expression data and their pathway level connections allows detection of the potential predictors that can prove to be putative therapeutic targets with biological and functional significance in progression of prostate cancer.

## 1 Introduction

### 1.1 The Prostate Cancer

Prostate Cancer (PC) is the second most widespread disease among men in the world and most often identified cancer in developing countries [1]. An exocrine gland, prostate discharges its secretions outside the male reproductive system. The liquidity of semen in prostate gland is maintained by the epithelial cells which produce a protein named PSA (prostate-specific antigen), which when high i.e. above 4 ng/ml is an indication of prostate cancer [2]. In most cases, the cancer starts in the gland cells that make the prostate fluid; such types of cancer are called adenocarcinoma. In fact, nearly 60% of all prostate cancers are diagnosed in men over the age of 65. As indicated by the rates of diagnosis, age is the biggest but not the only risk factor for prostate cancer. The major risk factors include age, family history, genetic factors, race, lifestyle, and dietary habits. The slow advancement of PC is witnessed through the changes in shape and size of the gland cells named as prostatic intraepithelial neoplasia (PIN). The possible complications include metastasis, incontinence and impact of changes of certain metabolic factors. Hormone therapy, that maintains low testosterone level in the body is the procedure to slow down the growth of already, spread PC, but it is often less effective. When the cancer grows with this therapy it is termed as castrate-resistant prostate cancer (CRPC).

### 1.2 Key Pathways in PC

The high variability in the disease progression and the paucity of the biomarker research provides incomplete understanding for the treatment of this disease. The challenge is to target prostate cancer biomarkers to address the unmet clinical needs in prostate cancer management [3]. There is urgent need to translate biologically relevant information and generate concordant results from different methods and procedures. For the identification of biomarkers, many studies have been performed, but to achieve clinical utility, these biomarkers should have a prognostic significance [4], [5]. Experiments using microarray technology have identified the expression of the genes associated with the different stages of PC in semen, tissues and glands [6]. Prostate cancer cells, like normal prostate cells, necessitate androgens to grow and survive. To develop successful future therapies, it is necessary to understand the critical events and complexities of AR signaling in the progression to CRPC. Significant evidence supports the fact that the occurrence and development of CRPC is casually related to continued transactivation of AR [7]. Many attempts have been made to search for gland-specific molecules those might help as potent biomarkers or as therapeutic agents due to the limitations in the standard treatment procedures of PC. Although, AR is an essential player that controls different elements in all phases of prostate carcinogenesis, but many other signaling pathways along with their interactions with AR signaling, are also critically implicated especially in advanced stages of prostate cancer. We have studied the expression profiles of the PC patients regarding their role in AR signaling pathways.

The already recounted increase in AR levels in 65% of CRPC cases was not alone overly significant; but, when combined with stabilization through c-Jun and phosphorylation from the MAPK cascade, the small increase becomes highly significant with increased activity [8]. This explains the correlation that exists between MAPK levels in the nucleus and levels of AR found in tumours.

### 1.3 Related Works

Studies probing MAPK activity in PC material suggested MAPK activity relates to development of a progressively complex and hormone independent PC. Preclinical studies have suggested a direct connection between the AKT which is a target for many anti-cancer agents [9] and AR signaling cascade, showing a dynamic interplay between these pathways during the development of ADT resistance and the development of androgen insensitivity. AKT signaling pathway has revealed a very important node that directs ADT resistance and stimulates tumour growth by adjusting the castrate levels of testosterone [10]. In addition, this pathway is altered at the genomic as well as the transcriptional level in almost all advanced PCs. Recently, a mathematical model has been developed to examine the steady-state and dynamic characteristics of the major feedback loops that synchronize the cross-talk between insulin-AKT and MAPK/ERK signaling pathways [11].

**Figure 1:**
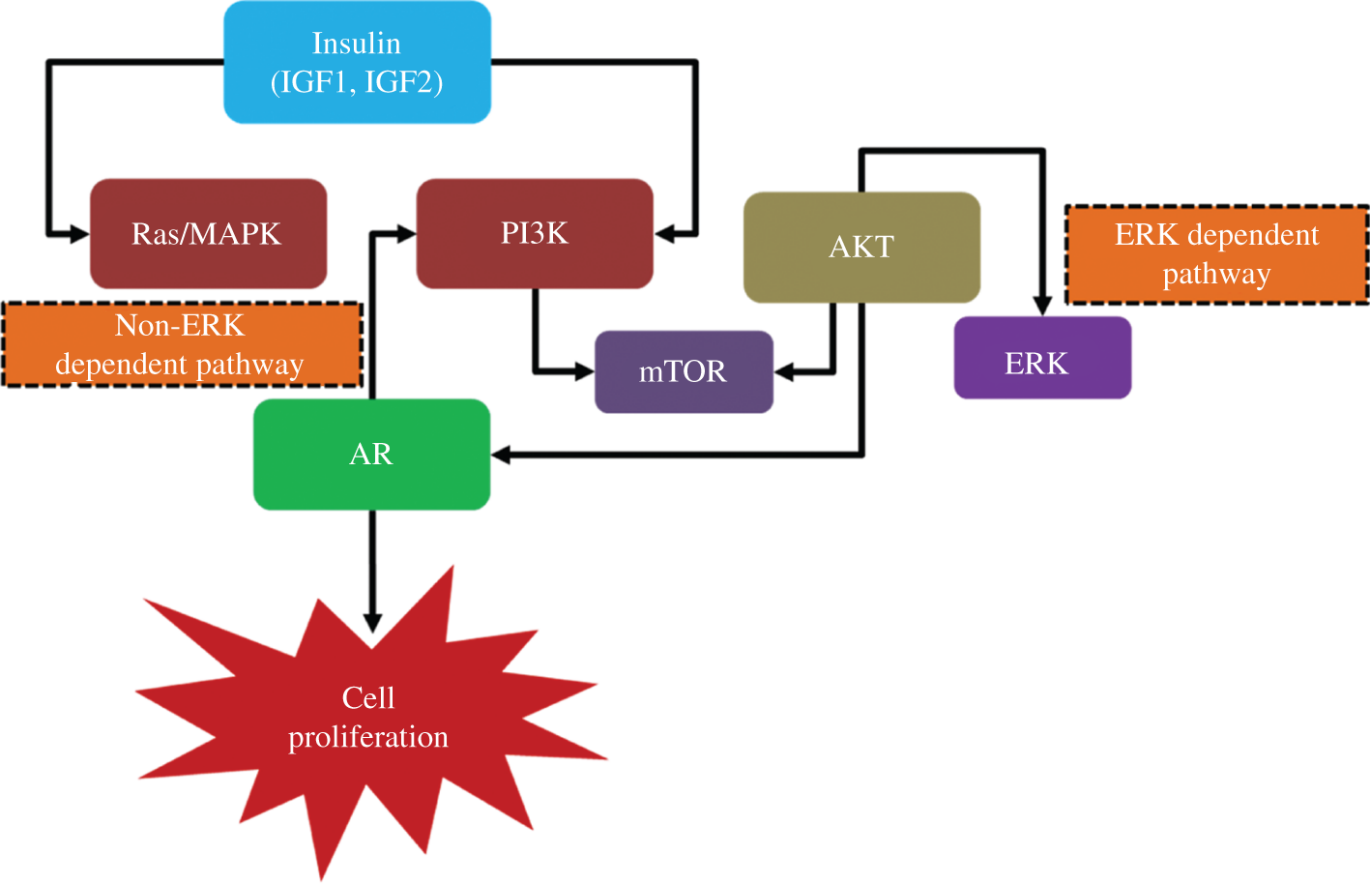
The interplay between AR, mTOR and MAPK signaling pathways.

Co-targeting strategies strive to improve cancer outcomes by combining therapies that inhibit driver genes in AR, MAPK, and mTOR pathways and are of great interest, since they are among the most frequently altered [10]. In our work we studied the interactions between three pathways AR, MAPK and mTOR (Figure 1) to conduct a cross-pathway examination for meta-analysis of PC related microarray data. Figure 1 depicts the cross regulatory effect of three pathways that shows cell proliferation (in terms of cancer progression) either in ERK or non-ERK dependent manner. These pathways represent close association thus showing interdependent pathway regulation as PI3K, AKT has regulatory effect on mTOR and likewise AKT, AR, and PI3K shows cross regulating effect. We approached the problem of computational data analysis using tools to integrate gene-specific expression changes in PC related samples and obtained several genes which can be targeted for detailed analysis and experimental procedures as therapeutic agents.

## 2 Materials and Methods

### 2.1 Data Collection

**Figure 2:**
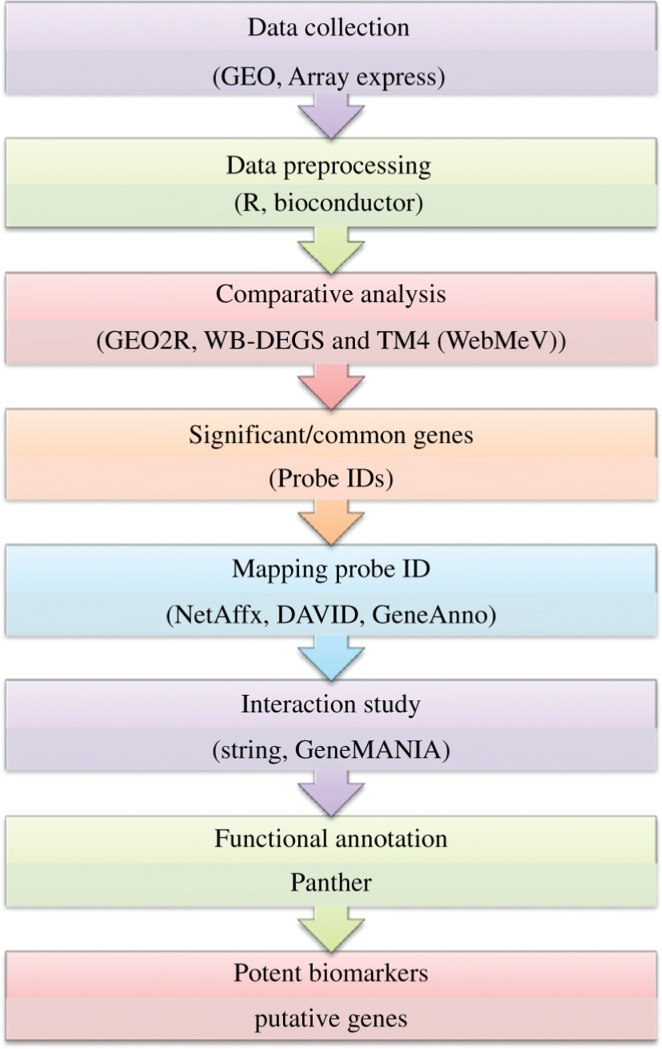
Flowchart of the meta-analysis performed implementing three pathways (AR, mTOR and MAPK).

The gene expression data have been retrieved from the National Centre for Biotechnology Information (NCBI); Gene Expression Omnibus (GEO) [12] and ArrayExpress [13]. Summarized methodology for the analysis is shown in Figure 2.

The information regarding the data sets that we incorporated in our microarray data analysis for all the three pathways is shown in Supplementary Table 1. We collected the gene expression data using the GEO series ID’s obtained from PubMed using the keyword search *“Prostate Cancer” AND “AR Pathway” AND “Homo Sapiens”* which resulted in 27 entries. The number of series datasets selected for AR pathway studies in prostate cancer is five, respectively (Supplementary Table 1).

### 2.2 Data Preprocessing

The processed gene expression data corresponding to these ID’s was downloaded from ArrayExpress to identify the differentially expressed genes within DNA repair pathways.

### 2.3 Comparative Analysis

Additionally, the same data was downloaded from GEO database in CEL format for further analysis. The ArrayExpress data was processed using WebMeV (Multiple Experiment Viewer) which is a free and open-source cloud service platform that supports analysis, visualization, and stratification of large genomic data, particularly for RNASeq and microarray data [14]. GeneSD (standard deviation) was done in the range 0–0.998 showing different colours in the heat map for top 20 probes/genes. The genes are classified based on the standard deviation of their expression values for all samples.

Similarly, GeneMAD (median of the absolute deviation) was performed; it is better at removing random clusters of multiple outliers of the expression values in the range 0–0.994, depicted by different colours in the heatmap. Principal component analysis (PCA) which is used for clustering large number of genes in complex biological networks was carried out on the same data in which each dot represents a PC sample plotted against its expression levels for the probes/genes. Different clustering method like k-means clustering was implicated using Euclidean distance measure methods. GEO2R was used for the expression analysis to compare two or more groups of samples, to distinguish genes that are expressed differentially throughout experimental states. Based on literature information, the samples are grouped into test and control for diseased and non-diseased samples, respectively. Both GEO2R and ArrayExpress uses same method i.e. Benjamini–Hochberg method. The method is selected by default because it is the most commonly used adjustment for microarray data and provides a good balance between discovery of statistically significant genes and limitation of false positives.

The BH threshold is defined for pre-specified 0 < α < 1 as:

$${\text{TBH}} = {\text{max}}\left\{ {{P_{(i)}}:{P_{(i)}} \le \alpha {i \over m},{\text{ }}0 \le i \le m} \right\}$$

The GEO2R results provide information regarding probe ID, its *t*-value which is a measure of the difference in means taking into consideration of data spread and sample sizes, and smaller *p*-value than a predefined significance level (0.01), defines the gene as differentially expressed. A *p*-value (or probability value) is very helpful in determining the confidence in the result decision that is taken under the null hypothesis. If the *p*-value is small; it indicates that it is very unlikely that the data was generated according to the null hypothesis. The *p*-value can be calculated either by the *t*-test or normal distribution parametrically or by a permutation method. Since the gene expression data are generally not normally distributed, the permutation test method is recommended to compute the *p*-values. The *p*-value of a permutation test is the cumulative sum of the probability of the observed outcome and the probability of all more extreme outcomes. Where the test statistic is denoted by *S_i_* and the observed score for gene *i* by $s_i*$, the *p*-value corresponding to the observed score $s_i^*$ is.

$${p_{i }}=\mathop \sum \limits_{{s_{i > {s_{{i^*}}}}}} P({S_i} = {s_{i}}|{H_0})$$

For each gene, a test statistic and its corresponding *p*-value are calculated to determine the extent of differential expression. The B value i.e. the log-odd function for each gene which is differentially expressed between the two groups. After, then the fold change on a logarithmic scale (logFC) value is shown along with the gene symbol and its title. The F-statistic calculates overall test of significance for that gene by combining the t-statistics for all pairwise comparisons. Generally F-statistic is given by comparison of the two variances, s1 and s2, and dividing them. It tests the equality of the class means for a fixed gene as:

$$F = {{(n - k) \sum {n_i}{{({Y_{i.}} - {Y_{i..}})}^2}} \over {(k - 1) \sum ({n_i} - 1)s_i^2}}$$

### 2.4 The Significant Genes

From the results top 20 and bottom 20 genes i.e. the over-expressed and under-expressed genes, respectively were identified by sorting the *p*-value column in descending order and discarding the value except <0.01 and <0.05 (as defined earlier as the significance level of 99% and 95%, respectively). Comparative analysis was performed for the pre-processing of data followed by statistical analysis and mapping of over expressed and under expressed genes for the involved pathways using WB-DEGS (Within and Between Group Comparisons for Differentially Expressed Gene Selection), a R based platform which performs pre-processing, visualization, and genes selection with an accuracy to minimize the false positive rates using some classical methods of gene selection [15].

### 2.5 Gene Mapping and Interactions

The corresponding gene mapping were performed using these probe ID’s, first in NetAffx™ [16] which enumerates the probe sequences and the consensus sequence interrupted by the probe Ids also DAVID (based on another agglomeration approach, heuristic fuzzy multiple-linkage partitioning) [17] and GeneAnnot [18] (which links between Affymetrix arrays and the rich human gene annotations) were utilized for the purpose. Then, input was provided in STRING database for each pathway and the network was constructed [19]. The same analysis was obtained from GeneMANIA [20] which helps to generate genetic interactions and predict the function of the genes.

### 2.6 Gene Annotations

Finally the gene annotations are performed using Panther tool that follows statistical enrichment using Mann-Whitney test to determine whether any ontology class or pathway has numeric values that are nonrandomly distributed with respect to the entire list of values and is given as:

$${U_{i }} = {R_i} - {{{n_{i}}({n_{i}} + 1)} \over 2}$$

It checks that the probability of the functional category distribution was drawn randomly from the reference distribution.

## 3 Results and Discussion

### 3.1 The Datasets

**Figure 3:**
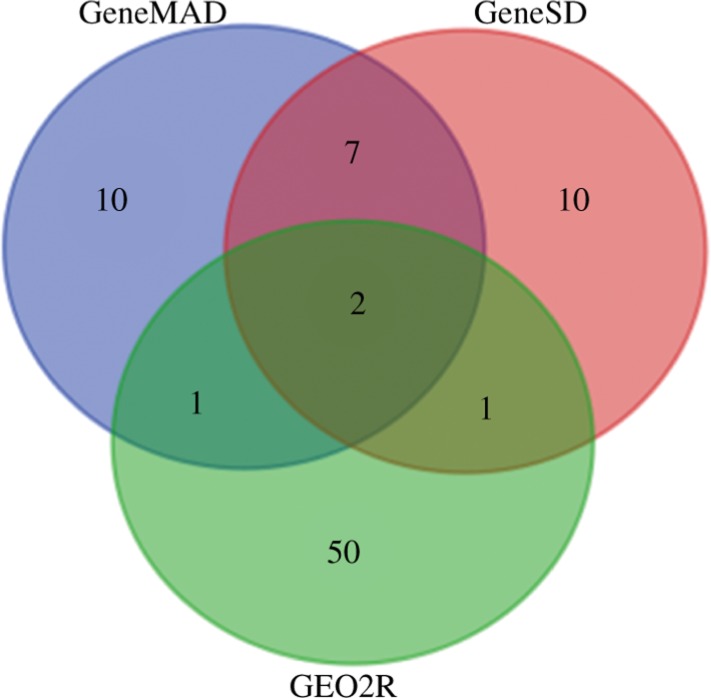
Common Genes in series GSE2443.

Analysis was performed for all series mentioned in Supplementary Table 1, as discussed above similar type of analysis has been performed for all the data; however here we are representing the detailed study for series GSE2443. The GEO2R analysis for GSE2443 gave a great number of genes but after applying the filters such as *p*-value cut-off we were left with only a few significant entities. The overlapping genes found may have some significance towards PC after analysing them through three different methods (i.e. GeneMAD, GeneSD, GEO2R). Through the analysis only two genes were identified to be the most common entities in all methods i.e. CEP57 (HGNC: 30794) and PDLIM5 (HGNC: 17468) (Figure 3).

**Figure 4:**
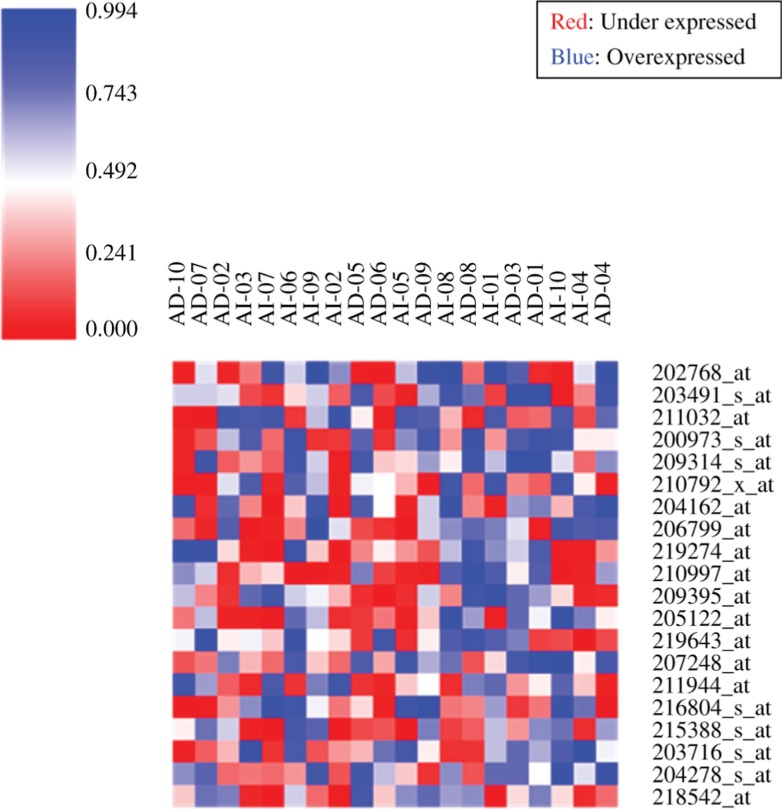
GeneMAD analysis for GSE2443 (20 samples), representative heatmap with red color showing under expressed probes and the blue ones being overexpressed.

**Figure 5:**
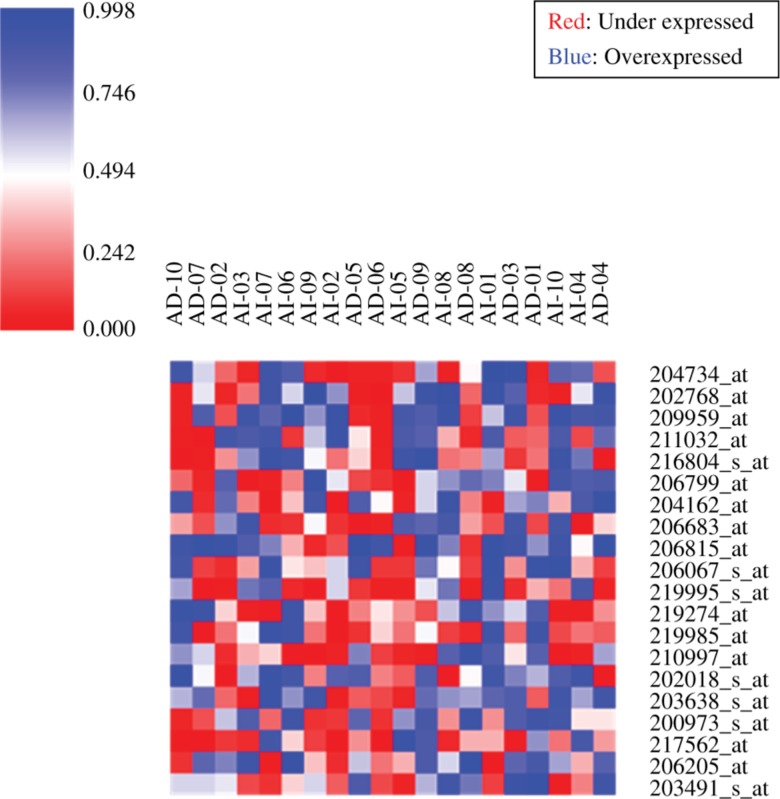
GeneSD analysis for GSE2443 (20 samples), representative heatmap with red color showing under expressed probes and the blue ones being overexpressed.

GeneMAD and GeneSD revealed the expression of probeIDs from 20 samples of series GSE2443 (Figure 4 and Figure 5). The range is between 0 and 0.998 for the expression values for each sample in the data set.

### 3.2 Identification of Set of Common Genes

**Figure 6:**
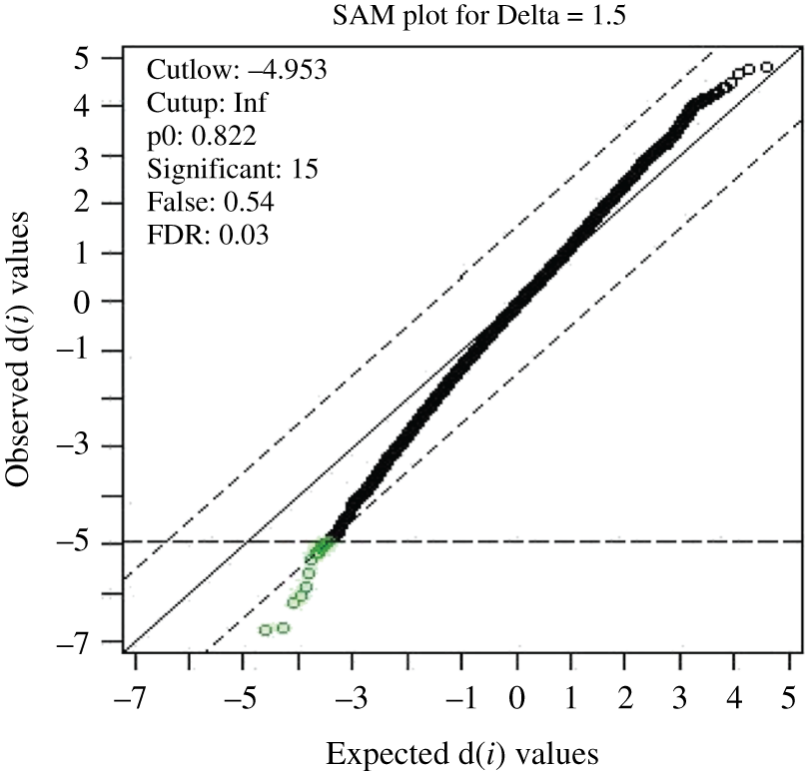
Between group comparisons in SAM analysis (GSE2443).

The genes which were found common between the microarray data analysis using GEO2R, WB-DEGS and TM4 (WebMeV) were tabulated and grouped differently. Then, input was provided in STRING database for each pathway and the network was constructed [19]. The interaction value between the two genes was considered greater than or equal to 0.9 which is considered significant and others are discarded. Then, using conditional formatting in excel, the genes were categorized into AR, MAPK and mTOR. The mapping of probeIDs to their respective genes was done using the supplementary file, containing the unabridged list of genes. The SAM analysis results at the delta value 1.5 gave 15 probes out of which 14 were mapped to the genes in NetAffx™ (Figure 6).

**Figure 7:**
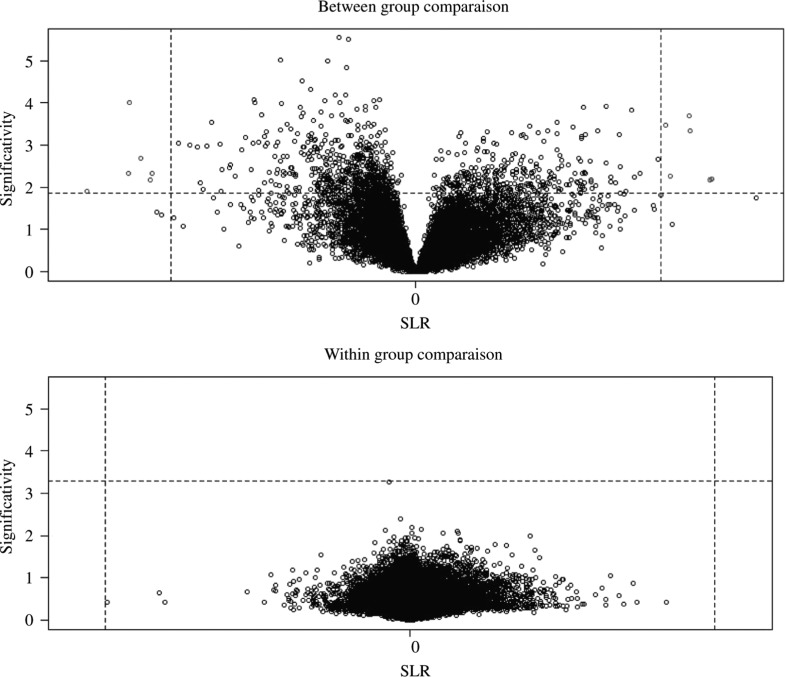
Simple Statistical Test (GSE2443) showing some significant range of the probes (11 probes) in between group comparison and none was found significant in within group comparison.

The Simple Statistical Test (paired *t*-test) gave 11 probes out of which 10 were successfully mapped to their respective genes (Figure 7). There was no significance in within group comparisons.

**Figure 8:**
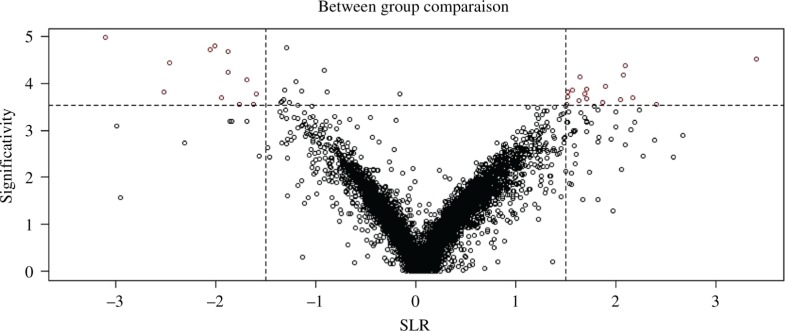
Twilight at logFC cutoff value of 1.5 for GSE2443.

The twilight statistical analysis at FC 1.5 gave 29 probes out of which 28 probes were mapped to genes (Figure 8). There was no significance in within group comparisons.

**Figure 9:**
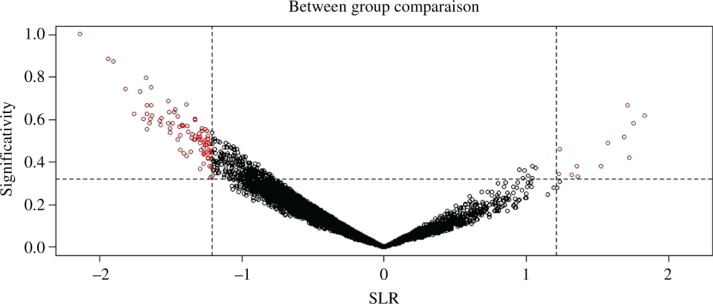
Linear models for GSE2443.

The linear model analysis gave a significant number of genes only in comparison between groups (Figure 9) but, the expressed genes showed the *p*-values greater than 0.01, which were not significant and thus were discarded.

Through similar procedures 8 genes were identified in series GSE8702 as *RPL23* (HGNC: 10316), *RPS9* (HGNC:10442), *DDC* (HGNC:2719), *KLK3* (HGNC:6364), *PLA2G2A* (HGNC:9031), *KLK2* (HGNC:6363), *CCL20* (HGNC:10619), *FAM198B* (HGNC:25312) (Supplementary Figures 1 and 2). Twenty seven were found in GSE21887 (Supplementary Figures 3 and 4). Again, in GSE33316, 13 genes were found based on the results (Supplementary Figures 5–8). In GSE67537 there were no common genes at any level. So, our analysis for Androgen receptor pathway (AR) ended up with 50 genes in total and only one gene in common across all the five series datasets. Now, for mitogen activated protein kinases pathway (MAPK), all four series datasets have given some common significant genes. In GSE20906 we found 11 genomic entities based on following results (Supplementary Figures 9–11). In GSE23038 we found 53 genes based on the following results (Supplementary Figures 12 and 13). In GSE29438 we found 4 significant genes based on the following results (Supplementary Figures 14 and 15). In GSE39735 156 significant genes were found based on the following results (Supplementary Figures 16–20).

So, our analysis for mitogen activated protein kinases pathway (MAPK) ended up with 224 genes in total with 9 genes in common across all the four series datasets. Now, for mechanistic target of rapamycin (m-TOR), all three series datasets have given a few more common significant genes. In GSE26332, 52 genes were found based on the following results (Supplementary Figures 21–23). In GSE49232 7 genes were found based on the following results (Supplementary Figures 24–26). In GSE32875 there were no genes in common between all the methods. So, our analysis for mechanistic target of rapamycin (m-TOR) ended up with 59 genes in total with 3 genes in common across all the three series datasets. Now, the genes obtained through all these three analysis were identified and were processed for functional annotation so that we can obtain few relevant genes. A total of 349 genes were identified in all pathways with 229 genes in MAPK, 63 genes in mTOR and 51 genes in AR pathway. After then the genes were categorized into the three pathways to visualize the interactions between them (Supplementary Table 2). The input of STRING was provided as these genes and the networks created for each pathway and multiple- pathway (in the pair of two). This determined the interactions between the genes belonging to same or different pathway.

### 3.3 The Gene Interactions and Annotations

From the network results obtained from STRING database, only, significant interactions having combined score greater than or equal to 0.9 was taken into consideration. The intra-pathway interactions include: AR-AR, mTOR-mTOR, MAPK-MAPK (Supplementary Table 2) and inter-pathway interactions include: AR-mTOR, mTOR-MAPK, AR-MAPK (Supplementary Table 3). The same analysis was obtained from GeneMANIA [20] which helps to generate genetic interactions and predict the function of the genes. Same type of input was provided with genes belonging to different pathways and the results were generated in the same format but with the weight value between the two genes. The weight represented the strength of interactions between them and this was processed by discarding the weights greater than 0.1 which is a significant interaction. The common genes inter-connections obtained from STRING and GeneMANIA are the putative genes which are suspected to play a major role in mediating the progression of the prostate cancer between the AR, mTOR and MAPK pathways (Table 1 and Table 2).

**Table 1:** Common intra-pathway connections between STRING and GeneMANIA for all three pathways.

AR-AR		mTOR-mTOR		MAPK-MAPK	
RPS20	RPS6	SEC11A	SPCS1	VAMP8	STX2
RPS8	RPS6	BNIP3	HIF1A	IL6	IL6R
RPL29	RPL23			SMN1	SMN2

**Table 2:** Common inter-pathway connections between STRING and GeneMANIA for all three pathways.

AR-mTOR		mTOR-MAPK	
IGFBP3	KLK3	IGFBP3	ADAM12
RPL29	EIF5	EDNRA	EDN1

The final genes that were found interconnected in three pathways are: *EDN1*(mTOR)*, EIF5*(MAPK)*, RPL23*(AR)*, RPL29*(AR)*, RPS6* (AR)*, RPS8*(AR)*, RPS20*(AR)*, PRKACB*(mTOR)*, SERBP1*(mTOR)*, NDUFA12*(mTOR)*, CDK1*(mTOR)*, EIF5B*(MAPK), and *UCHL5*(MAPK). Thus, the analysis finally leads to the 13 common genes. These genes are then further analysed based on their functional annotation to retrieve their importance in the regulation of prostate cancer in the AR, mTOR and MAPK pathway. Also, hubs were identified in the networks which are the genes which are connected to multiple genes; thereby influence the functions of each other (Table 3 and Table 4).

**Table 3:** List of Hub genes with their respective nodes across AR, MAPK and mTOR pathway.

Hub gene	Nodes
PRKACB	CALD1, CDK1, CDK6, CEP57, DHFR, EDN1
SERBP1	EEF2, EIF5, FAU, GNB4, KIAA0368, MNAT1
CDK1	NCOR1, NDUFB5, PRKACB, RAB11A, RPL23, RPL29, RPL31, RPL35A
EIF5B	RPL5, RPS10, RPS20, RPS29, RPS6, RPS8

**Table 4:** Gene description of the common genes found in all three pathways.

Gene symbol	Gene name	HGNC symbol	Panther family/subfamily	Panther protein class	UniProtKB
CDK1	Cyclin dependent kinase 1	1722	Cyclin-dependent kinase 1 (PTHR24056)	Non-receptor serine/threonine protein kinase(PC00167); non-receptor tyrosine protein kinase(PC00168)	P06493
EDN1	Endothelin 1	3176	Endothelin-1 (PTHR13874)		P05305
EIF5	Eukaryotic translation initiation factor 5	3299	Eukaryotic translation initiation factor 5 (PTHR23001)	G-protein modulator (PC00022); translation initiation factor(PC00224)	P55010
EIF5B	Eukaryotic translation initiation factor 5B	30793	Eukaryotic translation initiation factor 5B (PTHR43381)	G-protein(PC00020); hydrolase(PC0012); translation elongation factor(PC00222);translation initiation factor(PC00224)	O60841
NDUFA12	NADH:ubiquinone oxidoreductase subunit A12	23987	NADH dehydrogenase [ubiquinone] 1 alpha subcomplex subunit 12 (PTHR12910)		Q9UI09
PRKACB	Protein kinase cAMP-activated catalytic subunit beta	9381	Camp-dependent protein kinase catalytic subunit beta (PTHR24353)		P22694
RPL23	Ribosomal protein L23	10316	60S Ribosomal protein L23 (PTHR11761)	Ribosomal protein(PC00202)	P62829
RPL29	Ribosomal protein L29	10331	60S Ribosomal protein L29 (PTHR12884)	Ribosomal protein(PC00202)	P47914
RPS20	Ribosomal protein S20	10405	40S Ribosomal protein S20 (PTHR11700)	Ribosomal protein(PC00202)	P60866
RPS6	Ribosomal protein S6	10429	40S Ribosomal protein S6 (PTHR11502)		P62753
RPS8	Ribosomal protein S8	10441	40S Ribosomal protein S8 (PTHR10394		P62241
SERBP1	Serpine1 mRNA binding protein 1	17860	Plasminogen activator inhibitor 1 RNA-binding protein (PTHR12299)	RNA binding protein(PC00031)	Q8NC51
UCHL5	Ubiquitin C-terminal hydrolase L5	19678	Ubiquitin carboxyl-terminal hydrolase isozyme L5 (PTHR10589)	Cysteine protease(PC00081)	Q9Y5K5

## 4 Conclusion

We identified in total 13 candidate genes through the comparative analysis of the results obtained from the three microarray data analysis tools (MeV, GEO2R and WB-DEGS) for three different pathways. The results that we presented substantiates our approach of meta-analysis of expression data and pathway analysis of the genes obtained to find the putative targets which have biological and functional significance in the progression of prostate cancer. We also performed the comparative analysis of the various tools that we used to find the overlapping genes in all the three targeted DNA repair pathways. We proposed few entities based on quantitative data compilation, after investigating their association with the disease as well their overall association with the three pathways by connecting and visualizing them at pathway level. Final set of 13 genes could work as an accompaniment for the basis of experimental validations. Identification of the connections of genes, gene hubs and their functions can be novel therapeutic targets after experimental verification. They are therefore, proposed as potent biomarkers for the prostate cancer and their involvement in AR, MAPK and mTOR DNA repair pathways.

## References

[1] Pakzad R, Mohammadian-Hafshejani A, Ghoncheh M, Pakzad I, Salehiniya H. The incidence and mortality of prostate cancer and its relationship with development in Asia. Prostate Int 2015;3:135–40. 10.1016/j.prnil.2015.09.001 PMC4685206 26779461

[2] Shtricker A, Shefi S, Ringel A, Gillon G. PSA levels of 4.0–10 ng/mL and negative digital rectal examination. Antibiotic therapy versus immediate prostate biopsy. Int Braz J Urol 2009;35:551–5; discussion 5–8. 10.1590/s1677-55382009000500006 19860933

[3] Prensner JR, Rubin MA, Wei JT, Chinnaiyan AM. Beyond PSA: the next generation of prostate cancer biomarkers. Sci Transl Med 2012;4:127rv3. 10.1126/scitranslmed.3003180 PMC3799996 22461644

[4] Murphy L, Watson RW. Patented prostate cancer biomarkers. Nat Rev Urol 2012;9:464–72. 10.1038/nrurol.2012.130 22750955

[5] Principe S, Jones EE, Kim Y, Sinha A, Nyalwidhe JO, Brooks J, et al. In-depth proteomic analyses of exosomes isolated from expressed prostatic secretions in urine. Proteomics 2013;13:1667–71. 10.1002/pmic.201200561 PMC3773505 23533145

[6] Velonas VM, Woo HH, dos Remedios CG, Assinder SJ. Current status of biomarkers for prostate cancer. Int J Mol Sci 2013;14:11034–60. 10.3390/ijms140611034 PMC3709717 23708103

[7] Lonergan PE, Tindall DJ. Androgen receptor signaling in prostate cancer development and progression. J Carcinog 2011;10:20. PMID: . 21886458 10.4103/1477-3163.83937 PMC3162670

[8] Mukherjee R, McGuinness D, McCall P, Underwood M, Seywright M, Orange C, et al. Upregulation of MAPK pathway is associated with survival in castrate-resistant prostate cancer. Br J Cancer 2011;104:1920–8. 10.1038/bjc.2011.163 PMC3111196 21559022

[9] Porta C, Paglino C, Mosca A. Targeting PI3K/Akt/mTOR signaling in cancer. Front Oncol 2014;4:64. DOI: 10.3389/fonc.2014.00064. PMC3995050 24782981

[10] Edlind MP, Hsieh AC. PI3K-AKT-mTOR signaling in prostate cancer progression and androgen deprivation therapy resistance. Asian J Androl 2014;16:378–86. 10.4103/1008-682X.122876 PMC4023363 24759575

[11] Arkun Y. Dynamic modeling and analysis of the cross-talk between Insulin/AKT and MAPK/ERK signaling pathways. PloS One 2016;11. DOI: 10.1371/journal.pone.0149684. PMC4773096 26930065

[12] Barrett T, Wilhite SE, Ledoux P, Evangelista C, Kim IF, Tomashevsky M, et al. NCBI GEO: archive for functional genomics data sets – update. Nucleic Acids Res 2013;41(Database issue):D991–5. 10.1093/nar/gks1193 PMC3531084 23193258

[13] Parkinson H, Sarkans U, Shojatalab M, Abeygunawardena N, Contrino S, Coulson R, et al. ArrayExpress – a public repository for microarray gene expression data at the EBI. Nucleic Acids Res 2005;33(suppl_1):D553–D5. 10.1093/nar/gki056 PMC540010 15608260

[14] Howe EA, Sinha R, Schlauch D, Quackenbush J. RNA-Seq analysis in MeV. Bioinformatics 2011;27:3209–10. 10.1093/bioinformatics/btr490 PMC3208390 21976420

[15] Kaissi O, Nimpaye E, Singh TR, Vannier B, Ibrahimi A, Ghacham AA, et al. Genes selection comparative study in microarray data analysis. Bioinformation 2013;9:1019–22. 10.6026/97320630091019 PMC3910358 24497729

[16] Liu G, Loraine AE, Shigeta R, Cline M, Cheng J, Valmeekam V, et al. NetAffx: Affymetrix probesets and annotations. Nucleic Acids Res 2003;31:82–6. 10.1093/nar/gkg121 PMC165568 12519953

[17] Huang DW, Sherman BT, Tan Q, Kir J, Liu D, Bryant D, et al. DAVID Bioinformatics Resources: expanded annotation database and novel algorithms to better extract biology from large gene lists. Nucleic Acids Res 2007;35:W169–75. 10.1093/nar/gkm415 PMC1933169 17576678

[18] Chalifa-Caspi V, Shmueli O, Benjamin-Rodrig H, Rosen N, Shmoish M, Yanai I, et al. GeneAnnot: interfacing GeneCards with high-throughput gene expression compendia. Brief Bioinform 2003;4:349–60. 10.1093/bib/4.4.349 14725348

[19] Szklarczyk D, Morris JH, Cook H, Kuhn M, Wyder S, Simonovic M, et al. The STRING database in 2017: quality-controlled protein–protein association networks, made broadly accessible. Nucleic Acids Res 2017;45:D362–D8. 10.1093/nar/gkw937 PMC5210637 27924014

[20] Warde-Farley D, Donaldson SL, Comes O, Zuberi K, Badrawi R, Chao P, et al. The GeneMANIA prediction server: biological network integration for gene prioritization and predicting gene function. Nucleic Acids Res 2010;38:W214–20. 10.1093/nar/gkq537 PMC2896186 20576703

