# Supplementary material for: Gene Expression Studies to Identify Significant Genes in AR, MTOR, MAPK Pathways and their Overlapping Regulatory Role in Prostate Cancer
Source: J Integr Bioinform. 2019 May 28;16(3):20180080. doi: 10.1515/jib-2018-0080 (PMC6798856; doi:10.1515/jib-2018-0080)
Supplement: Supplementary file 1 [file jib-16-20180080-s001.docx]

**Supplementary Table 1.** Series Datasets for all three pathways

| Pathway | Series | Significance in study | Samples (Test\Control) | Platform | PMID |
| --- | --- | --- | --- | --- | --- |
| Androgen Receptor (AR) | GSE2443 | Yes | 10 \ 10 | GPL96  (HG-U133A) | [16203770](https://www.ncbi.nlm.nih.gov/pubmed/16203770) |
|  | GSE8702 | Yes | 5 \ 5 | [GPL570](https://www.ncbi.nlm.nih.gov/geo/query/acc.cgi?acc=GPL570)  (HG-U133_Plus_2) | [18302219](https://www.ncbi.nlm.nih.gov/pubmed/18302219) |
|  | GSE21887 | Yes | 4 \ 4 | [GPL570](https://www.ncbi.nlm.nih.gov/geo/query/acc.cgi?acc=GPL570)  (HG-U133_Plus_2) | [20145136](https://www.ncbi.nlm.nih.gov/pubmed/20145136) |
|  | GSE33316 | Yes | 5 \ 5 | [GPL570](https://www.ncbi.nlm.nih.gov/geo/query/acc.cgi?acc=GPL570)  (HG-U133_Plus_2) | [22108827](https://www.ncbi.nlm.nih.gov/pubmed/22108827) |
|  | GSE67537 | No^1^ | 9 \ 12 | [GPL13667](https://www.ncbi.nlm.nih.gov/geo/query/acc.cgi?acc=GPL13667)  (HG-U219) | NA |
| Mitogen-Activated Protein Kinase (MAPK) | GSE23038 | Yes | 6 \ 6 | [GPL571](https://www.ncbi.nlm.nih.gov/geo/query/acc.cgi?acc=GPL571)  (HG-U133A_2) | [21297939](https://www.ncbi.nlm.nih.gov/pubmed/21297939),[20689556](https://www.ncbi.nlm.nih.gov/pubmed/20689556) |
|  | GSE39735 | Yes | 3 \ 3 | GPL6244  (HuGene-1_0-st) | [23571738](https://www.ncbi.nlm.nih.gov/pubmed/23571738) |
|  | GSE20906 | Yes | 2 \ 2 | GPL4133  Agilent-014850 Whole Human Genome Microarray 4x44K G4112F | [21622724](https://www.ncbi.nlm.nih.gov/pubmed/21622724) |
|  | GSE29438 | Yes | 8 \ 4 | GPL13635  Nimblegen Homo sapiens HG18 Expression Array (12x135k) | [22012618](https://www.ncbi.nlm.nih.gov/pubmed/22012618) |
| Mechanistic Target of Rapamycin  (m-TOR) | GSE26332 | Yes | 1 \ 1 | [GPL570](https://www.ncbi.nlm.nih.gov/geo/query/acc.cgi?acc=GPL570)  (HG-U133_Plus_2) | [21212412](https://www.ncbi.nlm.nih.gov/pubmed/21212412) |
|  | GSE32875 | No^1^ | 4 \ 4 | GPL5175  (HuEx-1_0-st)  [GPL5188](https://www.ncbi.nlm.nih.gov/geo/query/acc.cgi?acc=GPL5188)  (HuEx-1_0-st) | [22194994](https://www.ncbi.nlm.nih.gov/pubmed/22194994) |
|  | GSE49232 | Yes | 2 \ 2 | [GPL570](https://www.ncbi.nlm.nih.gov/geo/query/acc.cgi?acc=GPL570)  (HG-U133_Plus_2) | [25670168](https://www.ncbi.nlm.nih.gov/pubmed/25670168) |

No correlation w.r.t p -value/log FC

**Supplementary Table 2.** Intra - pathway connections from STRING database

|  | | | | | | | | | | | | | | | | | |
| --- | --- | --- | --- | --- | --- | --- | --- | --- | --- | --- | --- | --- | --- | --- | --- | --- | --- |
| **AR-AR** | | | |  | | **mTOR-mTOR** | | | |  | | **MAPK-MAPK** | | | | | |
| RPL35A | | RPL23 | |  | | SEC11A | | SPCS1 | |  | | FAU | | RPL31 | | | |
| RPS20 | | RPL29 | |  | | NDUFA12 | | NDUFB5 | |  | | IL6R | | IL6 | | | |
| RPS29 | | RPS6 | |  | | EDN1 | | HIF1A | |  | | HSP90B1 | | LRP1 | | | |
| RPS29 | | RPS10 | |  | | HIF1A | | HSP90AB1 | |  | | UCHL5 | | KIAA0368 | | | |
| RPS8 | | RPS6 | |  | | BNIP3 | | HIF1A | |  | | STX2 | | VAMP8 | | | |
| RPS8 | | RPS29 | |  | | PRKACB | | YWHAZ | |  | | NCOR1 | | KAT2B | | | |
| RPS10 | | RPS9 | |  | | BRK1 | | ACTR3 | |  | | CDH3 | | CDH1 | | | |
| RPS29 | | EEF2 | |  | | HSP90AB1 | | HSPA9 | |  | | C1D | | NCOR1 | | | |
| RPS6 | | RPL5 | |  | | CDK1 | | CALD1 | |  | | HSPA4 | | HSP90B1 | | | |
| RPS8 | | RPS10 | |  | | PRKACB | | GNB4 | |  | | FAU | | EIF5B | | | |
| RPS8 | | EEF2 | |  | | CDK1 | | PRKACB | |  | | DHFR | | MTR | | | |
| RPS8 | | RPL23 | |  | | PRKACB | | RAB11A | |  | | ST3GAL6 | | B4GALT1 | | | |
| RPS6 | | EEF2 | |  | | TOMM5 | | HSPA9 | |  | | CDH11 | | CDH3 | | | |
| RPL35A | | RPL29 | |  | | SRSF7 | | POLR2L | |  | | RCOR1 | | HSPA4 | | | |
| RPS20 | | RPS10 | |  | | EDN1 | | PRKACB | |  | | RPL31 | | EIF5B | | | |
| RPS20 | | RPS29 | |  | |  | |  | |  | | CDH11 | | CDH1 | | | |
| RPS20 | | RPL23 | |  | |  | |  | |  | | IRF6 | | EGR1 | | | |
| RPS20 | | RPL5 | |  | |  | |  | |  | | IRF6 | | KAT2B | | | |
| RPL23 | | RPL29 | |  | |  | |  | |  | | SMN2 | | SMN1 | | | |
| RPS20 | | RPS8 | |  | |  | |  | |  | | CDK19 | | NCOR1 | | | |
| RPS8 | | RPL5 | |  | |  | |  | |  | | PDE3A | | NT5E | | | |
| RPL23 | | RPL5 | |  | |  | |  | |  | | QPRT | | NT5E | | | |
| RPS29 | | RPS9 | |  | |  | |  | |  | | HIST1H2BK | | HIST1H2BD | | | |
| RPS6 | | RPS9 | |  | |  | |  | |  | |  | |  | | | |
| RPL35A | | RPL5 | |  | |  | |  | |  | |  | |  | | | |
| RPS20 | | RPS9 | |  | |  | |  | |  | |  | |  | | | |
| RPS6 | | RPS10 | |  | |  | |  | |  | |  | |  | | | |
| RPS29 | | RPL5 | |  | |  | |  | |  | |  | |  | | | |
| RPS20 | | RPS6 | |  | |  | |  | |  | |  | |  | | | |
| RPS20 | | EEF2 | |  | |  | |  | |  | |  | |  | | | |
| RPL5 | | RPL29 | |  | |  | |  | |  | |  | |  | | | |
| RPL5 | | EEF2 | |  | |  | |  | |  | |  | |  | | | |
| RPL23 | | RPS6 | |  | |  | |  | |  | |  | |  | | | |
| RPS8 | | RPS9 | |  | |  | |  | |  | |  | |  | | | |
| RPS29 | | RPL23 | |  | |  | |  | |  | |  | |  | | | |
| RPS8 | | RPL29 | |  | |  | |  | |  | |  | |  | | | |
| RPL5 | | RPS10 | |  | |  | |  | |  | |  | |  | | | |
| RPS29 | | RPL29 | |  | |  | |  | |  | |  | |  | | | |
| RPL35A | | RPS8 | |  | |  | |  | |  | |  | |  | | | |
| RPL23 | | EEF2 | |  | |  | |  | |  | |  | |  | | | |
| RPL5 | | RPS9 | |  | |  | |  | |  | |  | |  | | | |
| EEF2 | | RPS9 | |  | |  | |  | |  | |  | |  | | | |
| RPS20 | | RPL35A | |  | |  | |  | |  | |  | |  | | | |
| RPL35A | | RPS6 | |  | |  | |  | |  | |  | |  | | | |
| RPL35A | | RPS29 | |  | |  | |  | |  | |  | |  | | | |
| RPS6 | | RPL29 | |  | |  | |  | |  | |  | |  | | | |
| RPS10 | | RPL29 | |  | |  | |  | |  | |  | |  | | | |
| RPS10 | | EEF2 | |  | |  | |  | |  | |  | |  | | | |
| RPL35A | | RPS10 | |  | |  | |  | |  | |  | |  | | | |
| RPL23 | | RPS10 | |  | |  | |  | |  | |  | |  | | | |
| RPL35A | | EEF2 | |  | |  | |  | |  | |  | |  | | | |
| RPL23 | | RPS9 | |  | |  | |  | |  | |  | |  | | | |
| EEF2 | | RPL29 | |  | |  | |  | |  | |  | |  | | | |
| RPL35A | | RPS9 | |  | |  | |  | |  | |  | |  | | | |
| RPS9 | | RPL29 | |  | |  | |  | |  | |  | |  | | | |
| CCL20 | | OPRK1 | |  | |  | |  | |  | |  | |  | | | |
| **Supplementary Table 3.** Inter - pathway connections from STRING database. | | | | | | | | | | | | | | | |  |  |
| **AR-mTOR** | | | |  | | **mTOR-MAPK** | | | |  | | **AR-MAPK** | | | |  |  |
| KLK3 | | IGFBP3 | |  |  | EDN1 | | EDNRA | |  |  | RPL23 | | FAU | |  |  |
| RPS6 | | SERBP1 | |  | | CDK1 | | MNAT1 | |  | | RPL29 | |  |  |  |  |
| RPS10 | |  |  |  | |  |  | CDK6 | |  | | EEF2 | |  |  |  |  |
| RPS29 | |  |  |  | |  |  | NCOR1 | |  | | RPS29 | |  |  |  |  |
| RPS20 | |  |  |  | |  |  | DHFR | |  | | RPS20 | |  |  |  |  |
| EEF2 | |  |  |  | | EDN1 | | IL6 | |  | | RPL5 | |  |  |  |  |
| RPS8 | |  |  |  | | EIF5 | | EIF5B | |  | | RPS8 | |  |  |  |  |
| RPS9 | |  |  |  | | SERBP1 | | FAU | |  | | RPS9 | |  |  |  |  |
| DDC | | MAOA | |  | | EIF5 | |  |  |  | | RPS10 | |  |  |  |  |
| CEP57 | | CDK1 | |  | | SEC11A | |  |  |  | | RPS6 | |  |  |  |  |
| NCAPD3 | |  |  |  | | SPCS1 | |  |  |  | | RPL35A | |  |  |  |  |
| CP | | HIF1A | |  | | HSP90AB1 | |  |  |  | | RPL23 | | RPL31 | |  |  |
| RPS6 | | EIF5 | |  | |  |  | TTC32 | |  | | RPL5 | |  |  |  |  |
| RPL35A | | EIF5 | |  | | GPX2 | | GGT1 | |  | | RPL35A | |  |  |  |  |
| RPL5 | | EIF5 | |  | | HIF1A | | HSPA4 | |  | | RPS6 | |  |  |  |  |
| RPS20 | | EIF5 | |  | |  |  | NT5E | |  | | RPL29 | |  |  |  |  |
| RPS8 | | EIF5 | |  | |  |  | NCOR1 | |  | | RPS10 | |  |  |  |  |
| RPS29 | | EIF5 | |  | | HSP90AB1 | | HSPA4 | |  | | RPS20 | |  |  |  |  |
| RPS10 | | EIF5 | |  | |  |  | AKT3 | |  | | RPS29 | |  |  |  |  |
| RPL29 | | EIF5 | |  | |  |  | HSPA13 | |  | | EEF2 | |  |  |  |  |
| RPL23 | | EIF5 | |  | | HSPA9 | | HSP90B1 | |  | | RPS8 | |  |  |  |  |
| RPS20 | | SPCS1 | |  | | IGFBP3 | | ADAM12 | |  | | RPS9 | |  |  |  |  |
| RPL35A | | SPCS1 | |  | | POLR2L | | MNAT1 | |  | | RPS8 | | EIF5B | |  |  |
| RPS10 | | SEC11A | |  | | PRKACB | |  |  |  | | RPL5 | |  |  |  |  |
| RPS29 | | SEC11A | |  | | EIF5 | | RPL31 | |  | | RPL23 | |  |  |  |  |
| NCAPD3 | | SET | |  | | SPCS1 | |  |  |  | | RPS20 | |  |  |  |  |
| RPS10 | | SPCS1 | |  | | SEC11A | |  |  |  | | RPS6 | |  |  |  |  |
| RPS6 | |  |  |  | | TKT | | RPIA | |  | | RPS29 | |  |  |  |  |
| RPS8 | |  |  |  | |  | |  | |  | | RPL29 | |  |  |  |  |
| RPL5 | |  |  |  | |  | |  | |  | | RPS10 | |  |  |  |  |
| RPL29 | |  |  |  | |  | |  | |  | | RPL35A | |  |  |  |  |
| RPS29 | |  |  |  | |  | |  | |  | | GDF15 | | EGR1 | |  |  |
| RPL23 | |  |  |  | |  | |  | |  | | LIFR | | IL6 | |  |  |
| RPL5 | | SEC11A | |  | |  | |  | |  | | RBMS1 | | SARNP | |  |  |
| RPS6 | |  |  |  | |  | |  | |  | | KLK3 | | CDK6 | |  |  |
| RPL29 | |  |  |  | |  | |  | |  | |  |  | EGR1 | |  |  |
| RPS20 | |  |  |  | |  | |  | |  | |  | |  | |  |  |
| RPL35A | |  |  |  | |  | |  | |  | |  | |  | |  |  |
| RPS8 | |  |  |  | |  | |  | |  | |  | |  | |  |  |
| RPL23 | |  |  |  | |  | |  | |  | |  | |  | |  |  |
| PLA2G2A | | CHPT1 | |  | |  | |  | |  | |  | |  | |  |  |


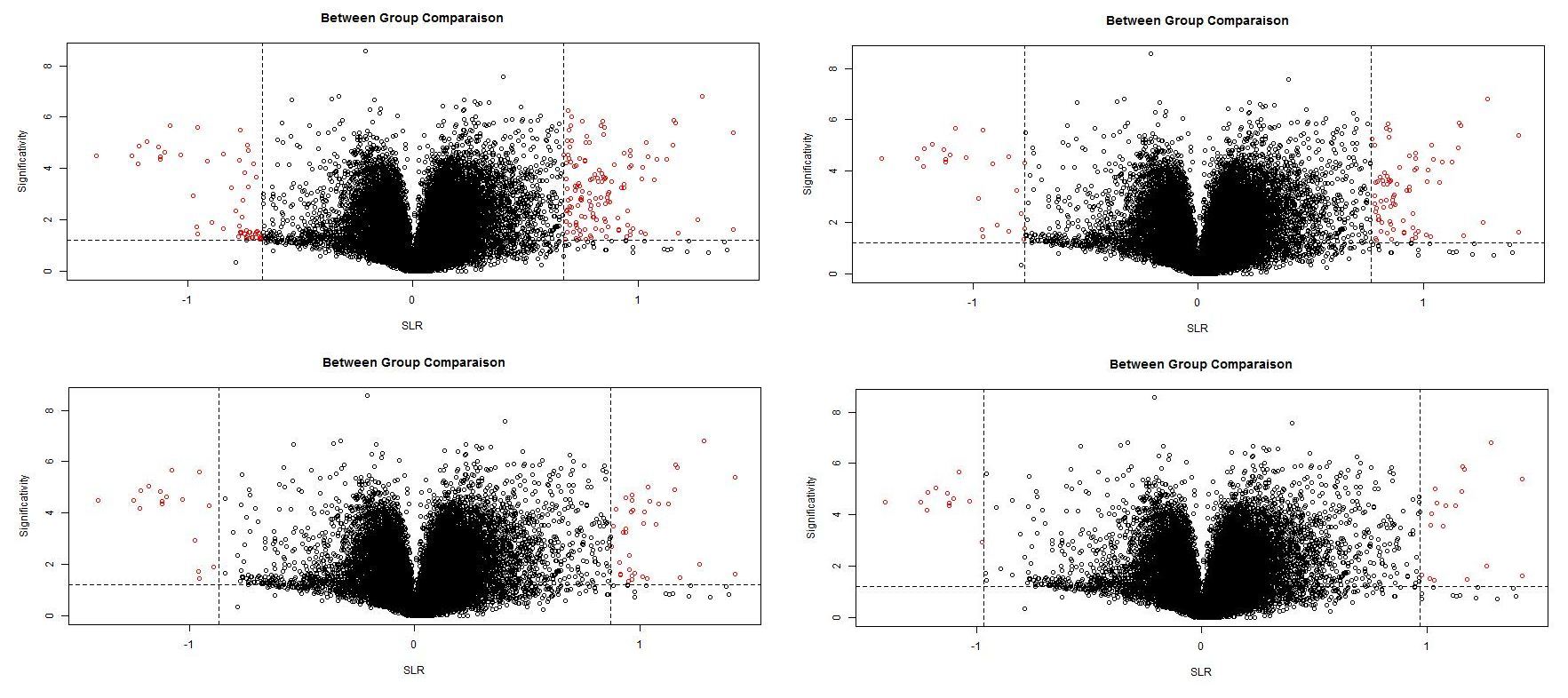


**Supplementary Figure 1.** Simple Statistical Test at logFC cutoff values 0.67 for GSE8702


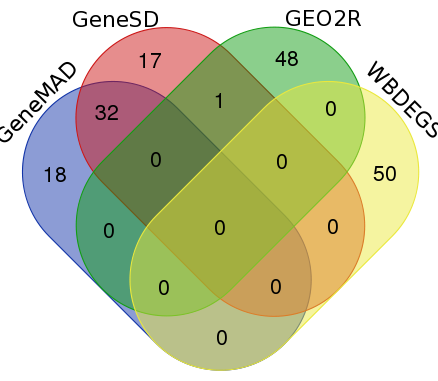


**Supplementary Figure 2.** Common genes obtained through GeneMAD, GeneSD, GEO2R, and WBDEGS based on logFC value for GSE8702


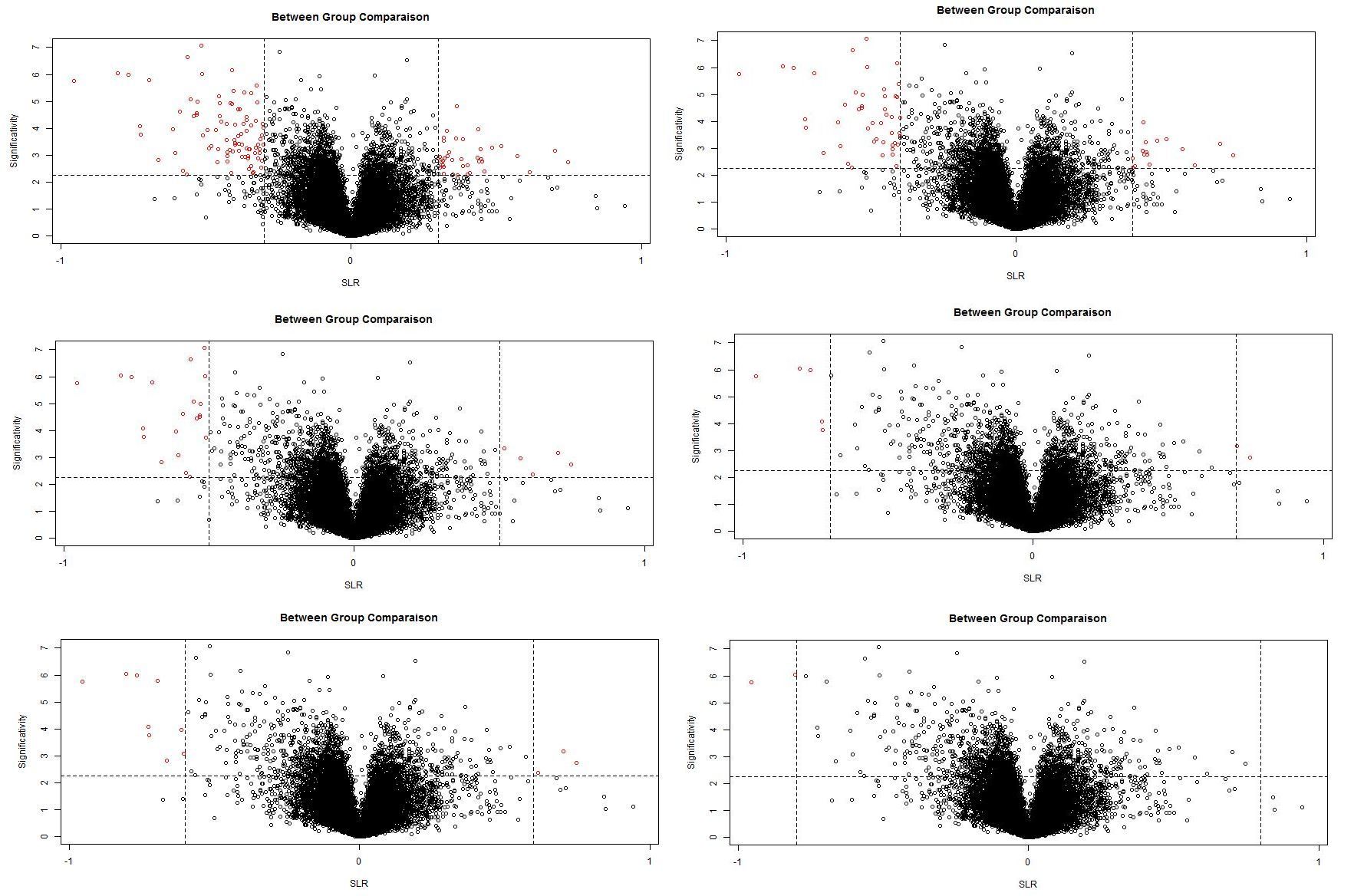


**Supplementary Figure 3.** Simple Statistical Test at logFC cutoff values 0.3 for GSE21887


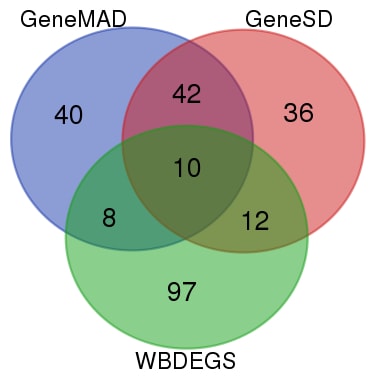


**Supplementary Figure 4.** Input of top 100 genes since p -value is less than 0.01 (bottom) for GSE21887


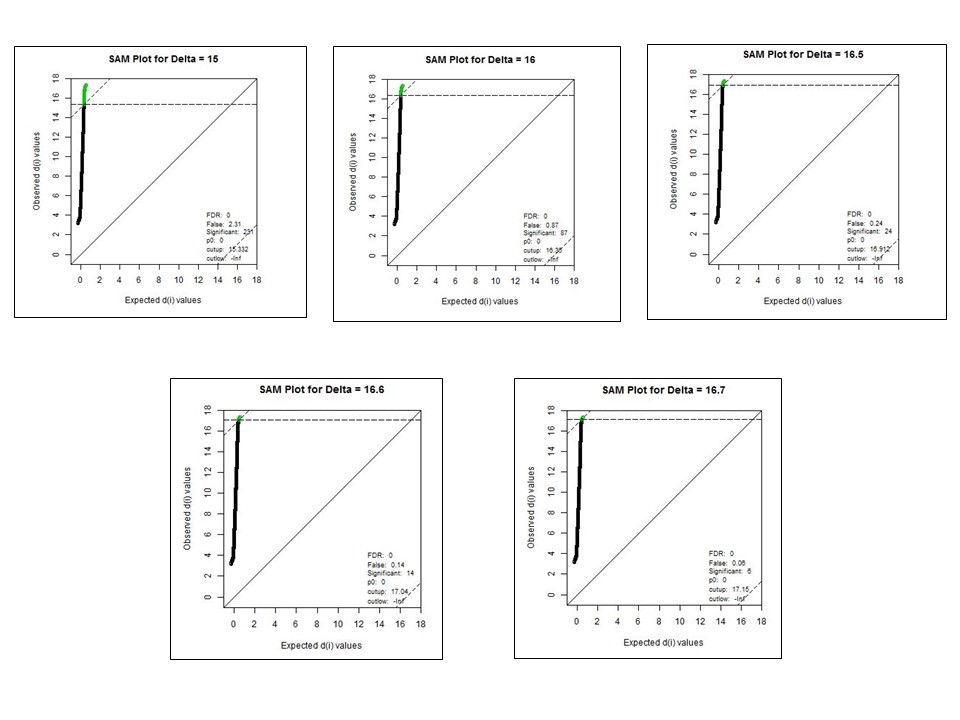


**Supplementary Figure 5.** SAM Analysis for GSE33316


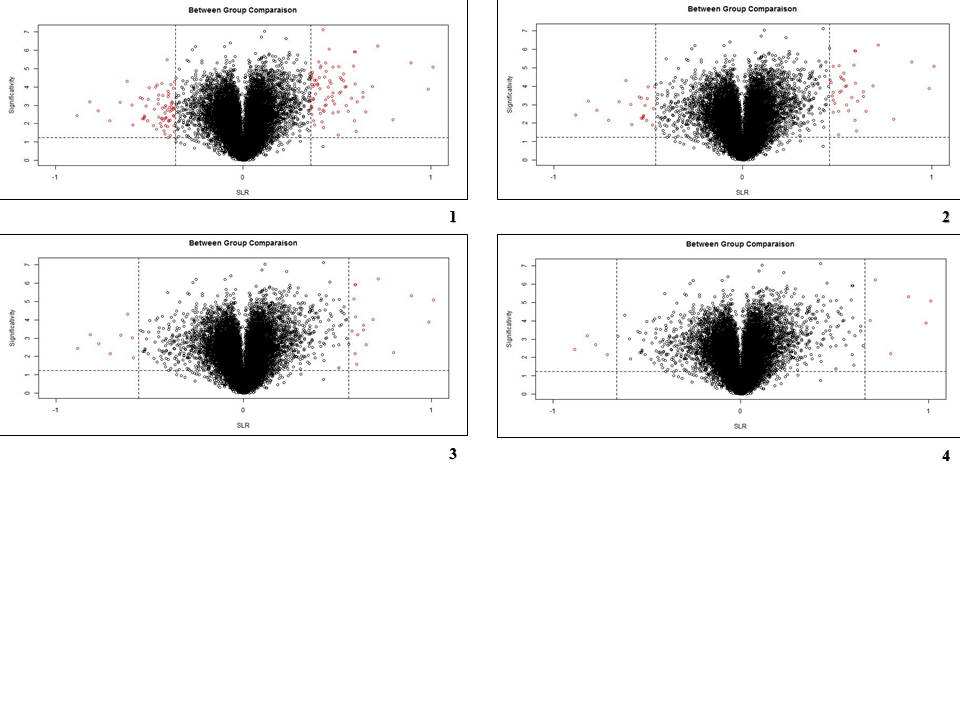


**Supplementary Figure 6.** Simple Statistical Test at log FC values 0.36 for GSE33316


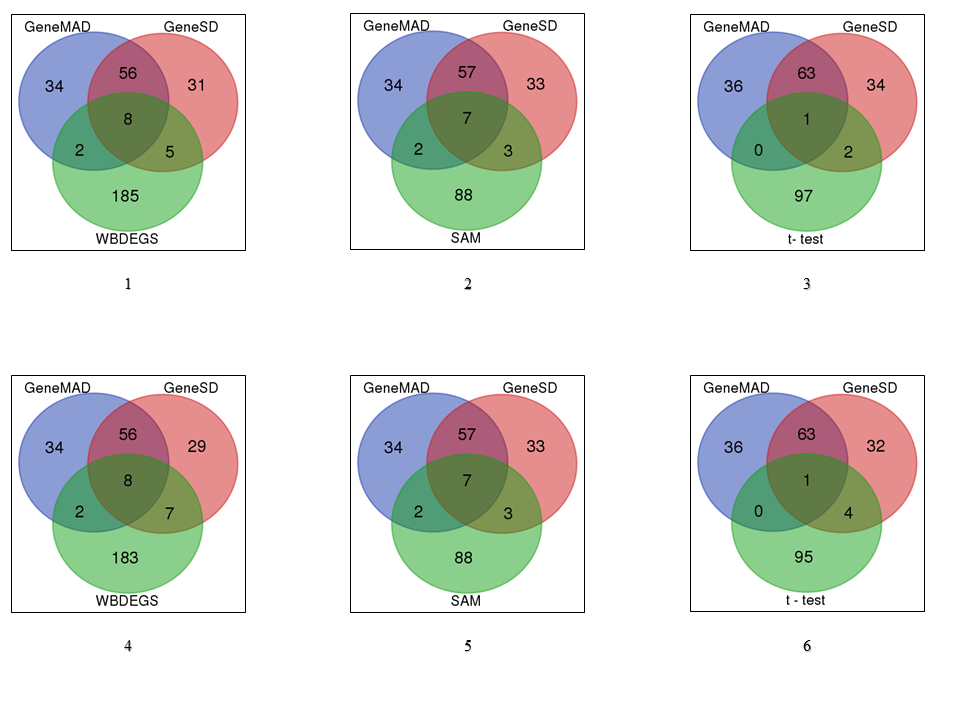


**Supplementary Figure 7.** p - value less than 0.01 for GSE33316


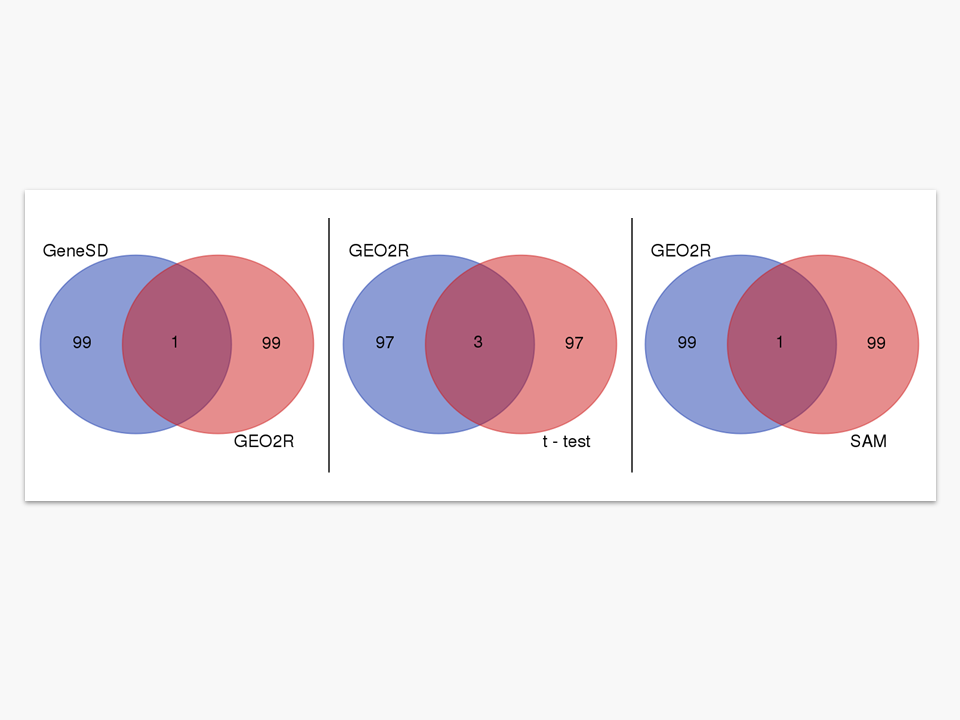


**Supplementary Figure 8.** Pair wise Comparisons of genes showing p - value less than 0.05 for GSE33316


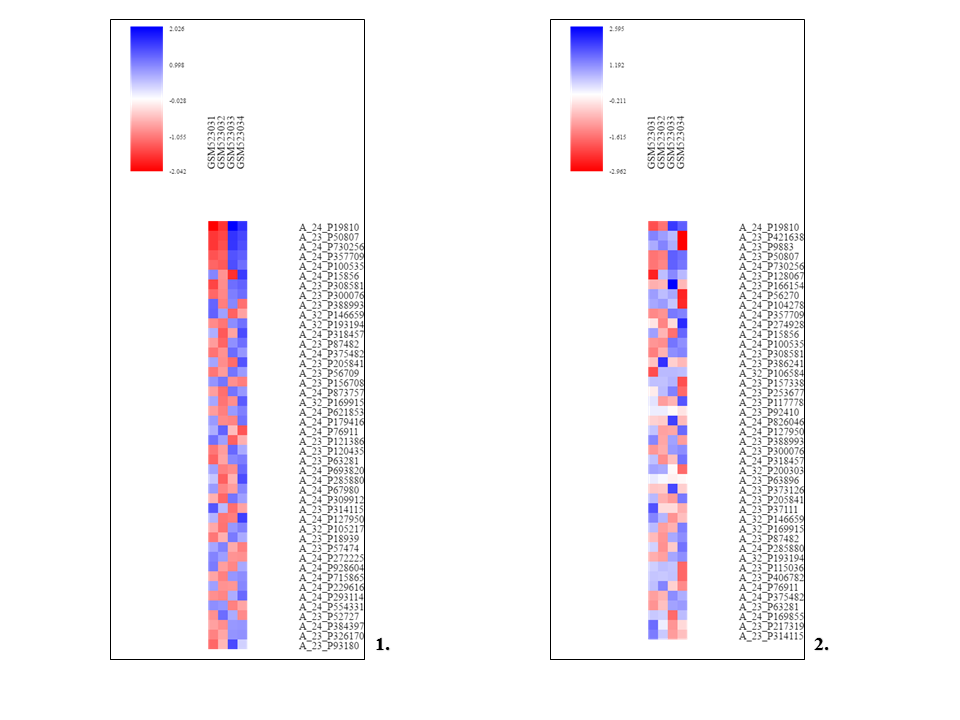


**Supplementary Figure 9.** Expression Levels using 1.) GeneMAD Top 50 2.) GeneSD Top 50 for GSE20906


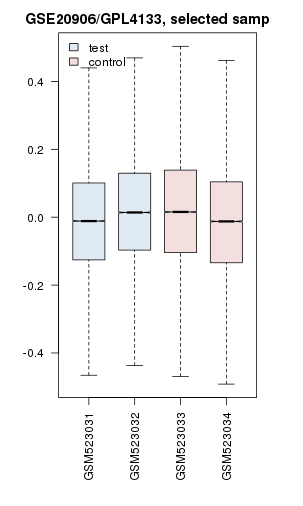


**Supplementary Figure 10.** Box and Whiskers Plot generated using GEO2R for GSE20906


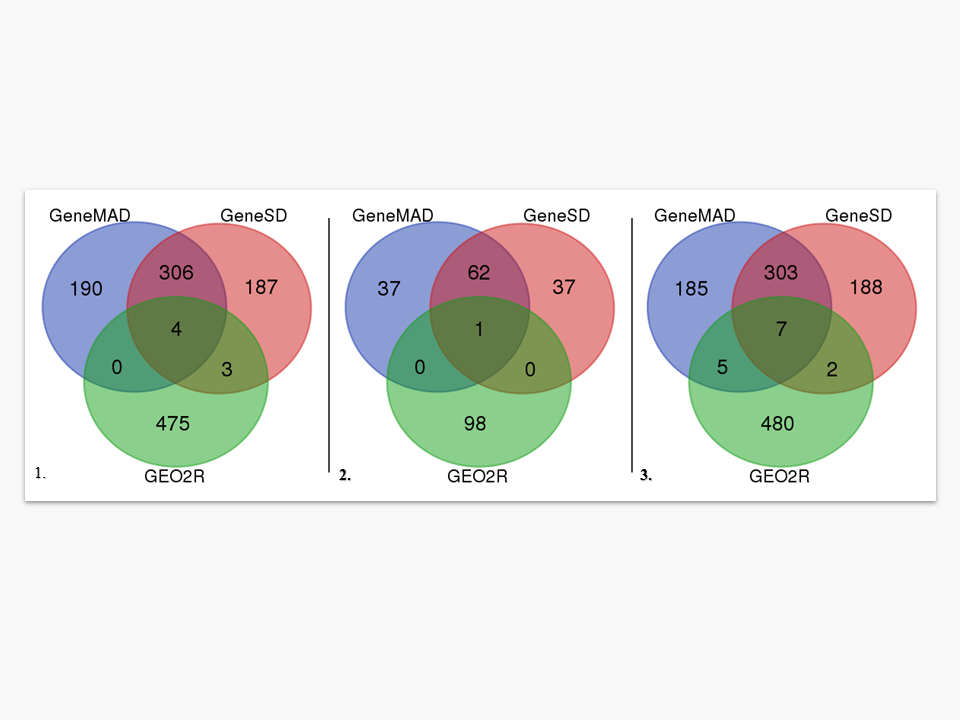


**Supplementary Figure 11.** Input of top 500 genes since p - value is less than 0.01 for GSE20906


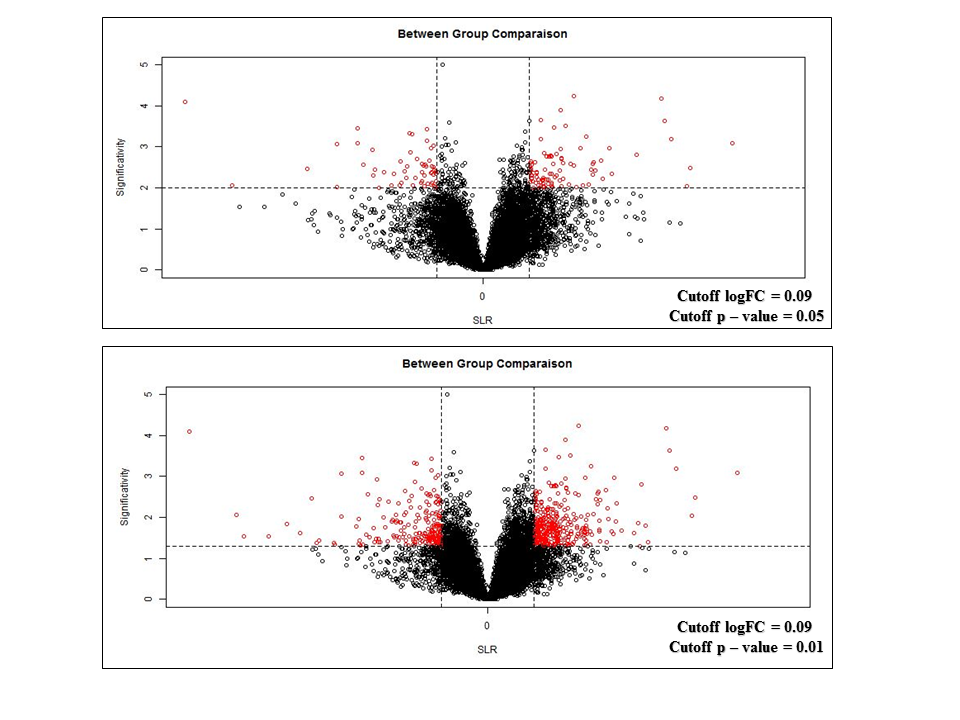


**Supplementary Figure 12.** Simple Statistical Test for GSE23038


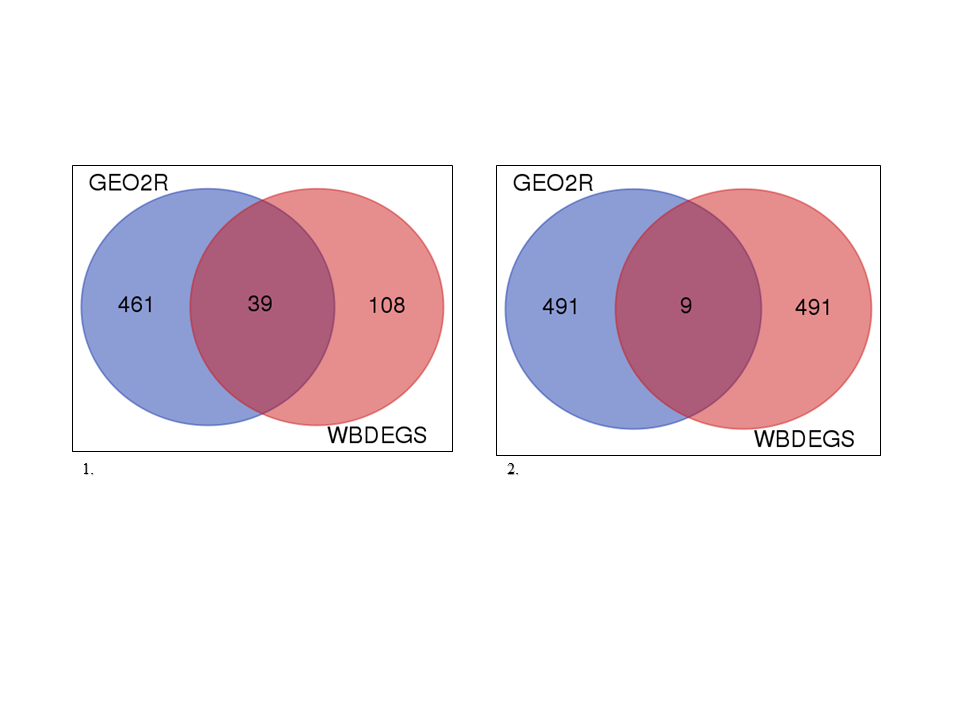


**Supplementary Figure 13.** Input of top 500 genes for . p – value less than 0.01 for GSE23038


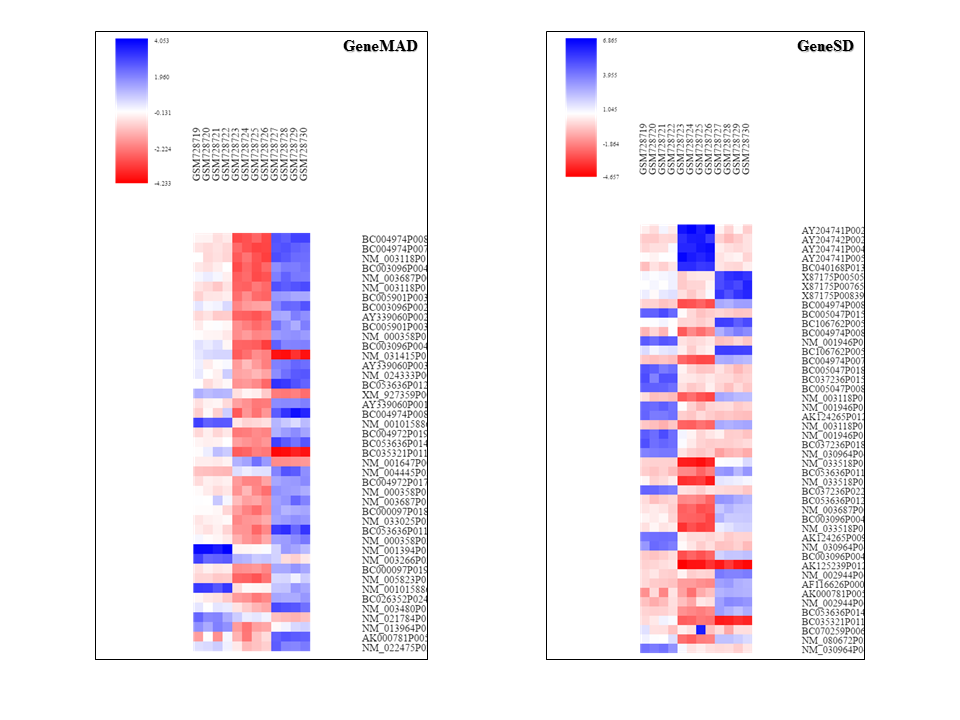


**Supplementary Figure 14.** WebMeV (TM4) Analysis for GSE29438


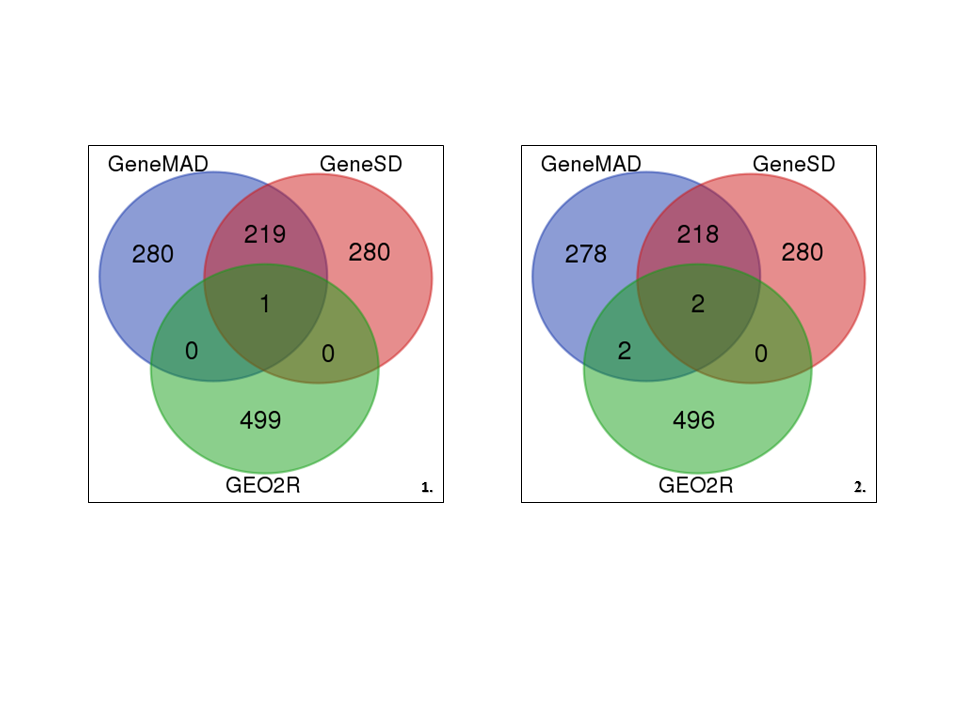


**Supplementary Figure 15.** Input of top 500 genes for p – value less than 0.01 GSE29438


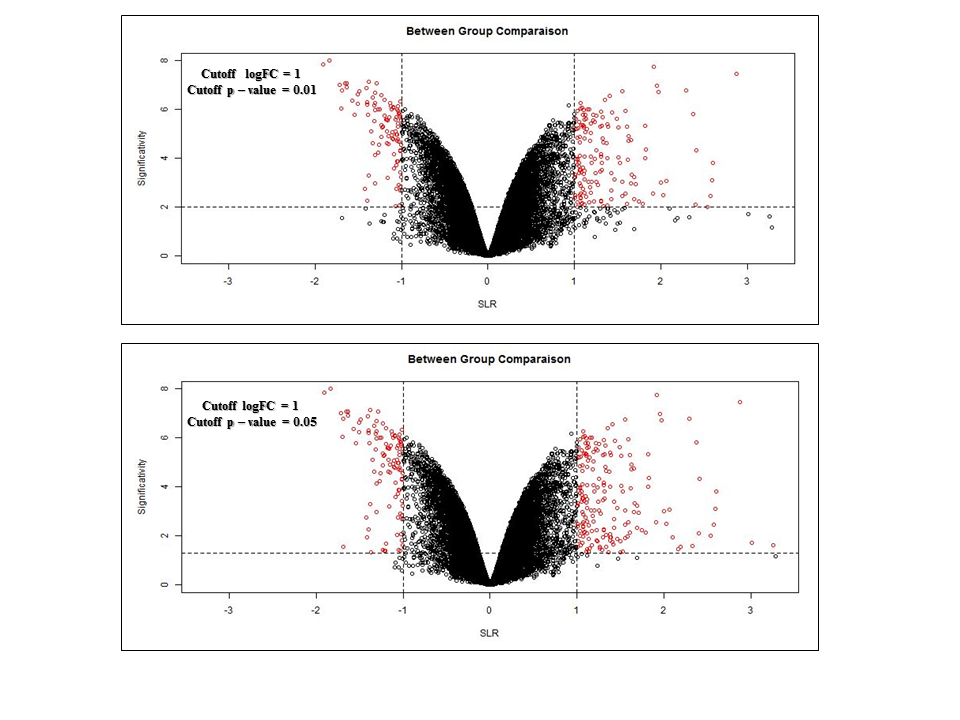


**Supplementary Figure 16.** Linear Models for GSE39735


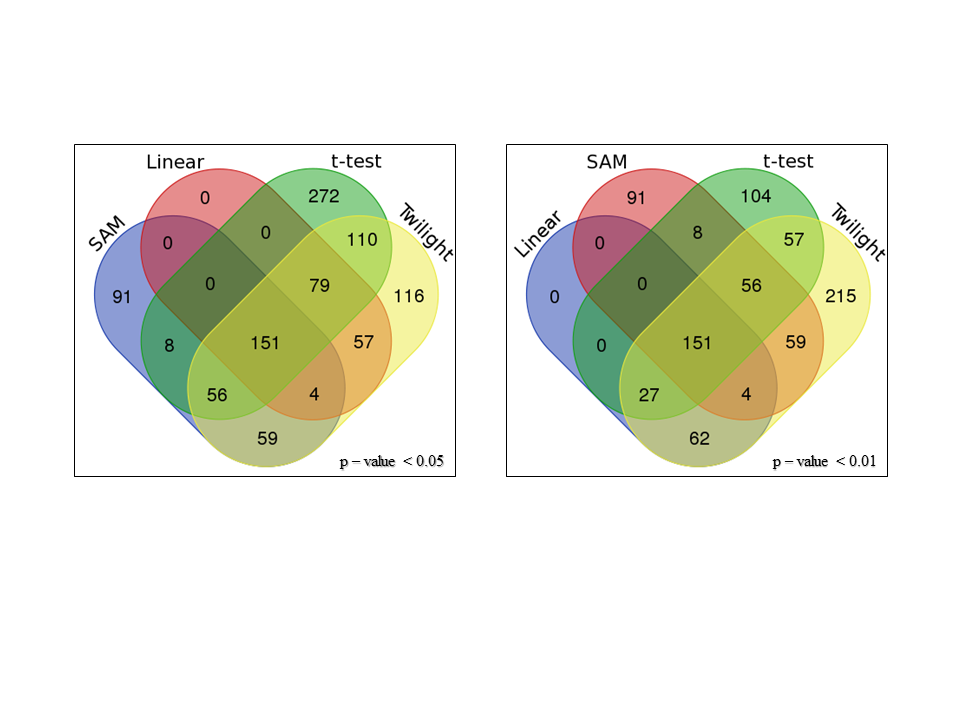


**Supplementary Figure 17.** Venn diagram based on p – values for GSE39735


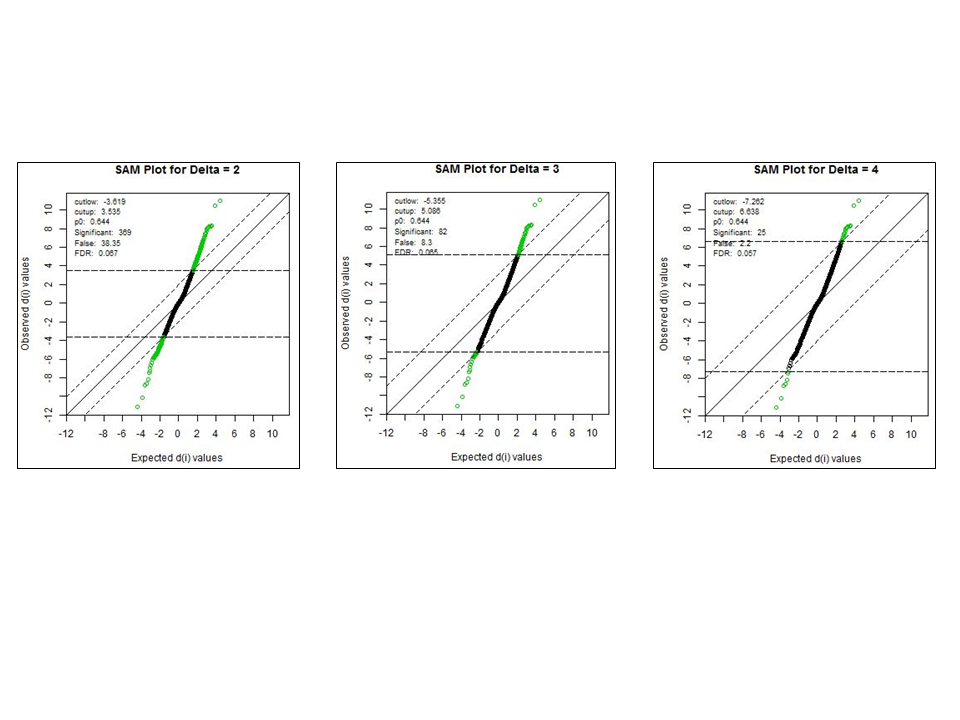


**Supplementary Figure 18.** SAM Analysis for GSE39735


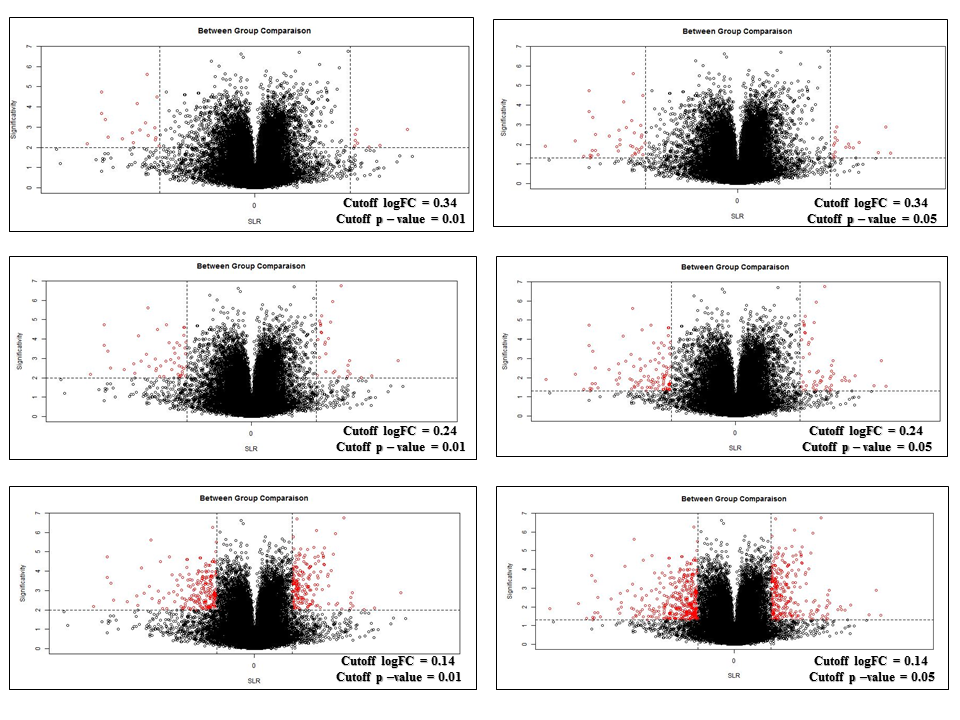


**Supplementary Figure 19.** Simple Statistical Test for GSE39735


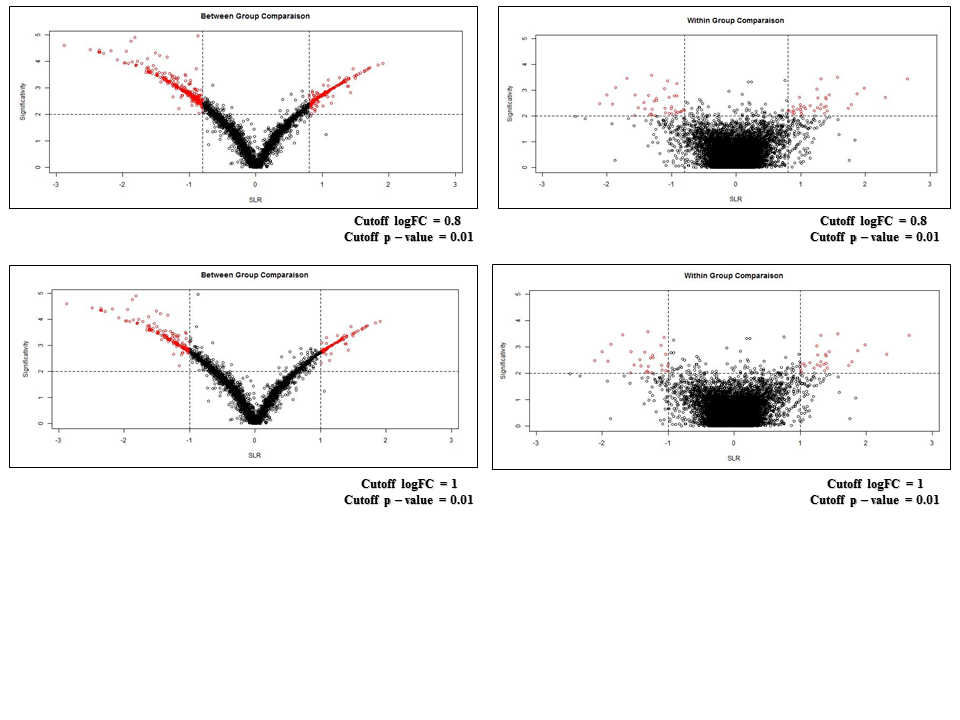


**Supplementary Figure 20.** Twilight Models for GSE39735


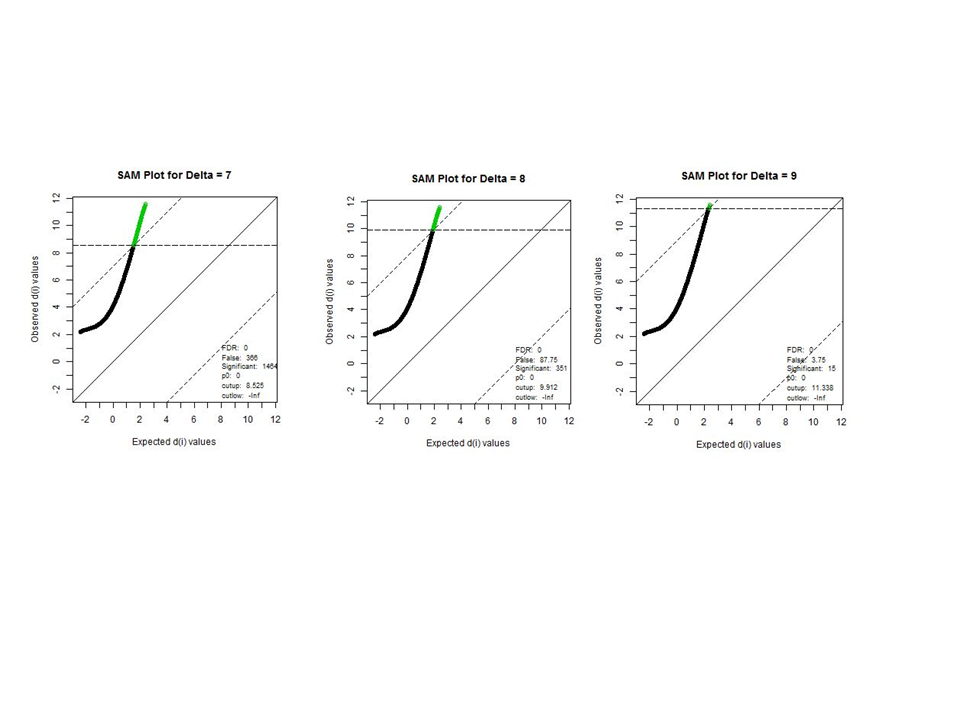


**Supplementary Figure 21.** SAM Analysis for GSE26332


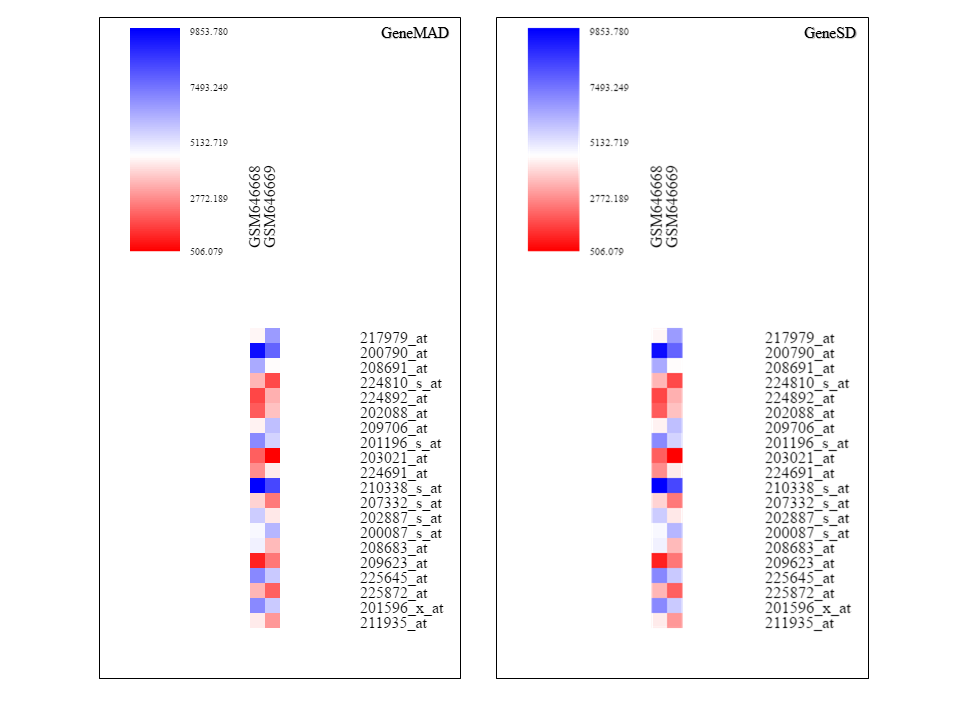


**Supplementary Figure 22.** WebMeV (TM4) Analysis for Top20 genes. The results for both GeneMAD and GeneSD are same due to single test and control samples in the dataset


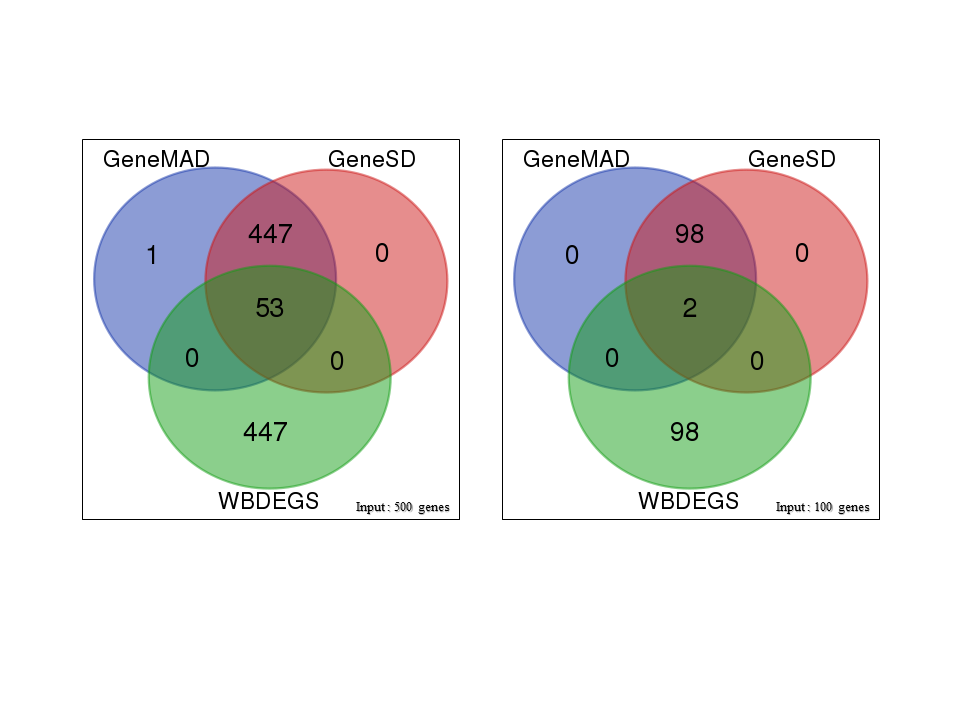


**Supplementary Figure 23.** Venn Diagram based on p - value < 0.01 for GSE26332


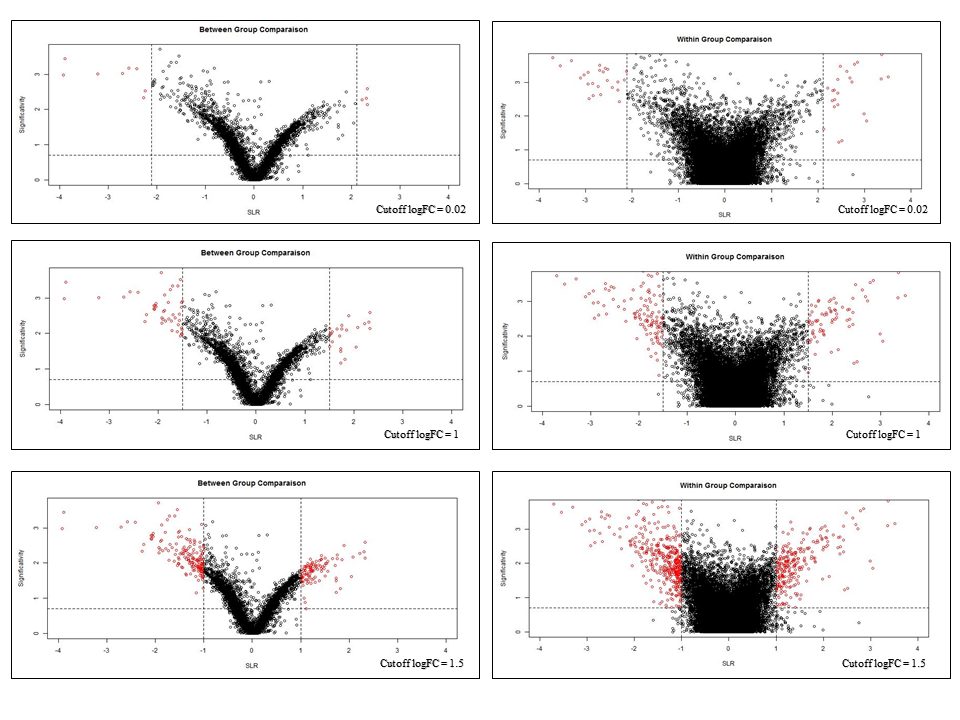


**Supplementary Figure 24.** Twilight Models for GSE49232


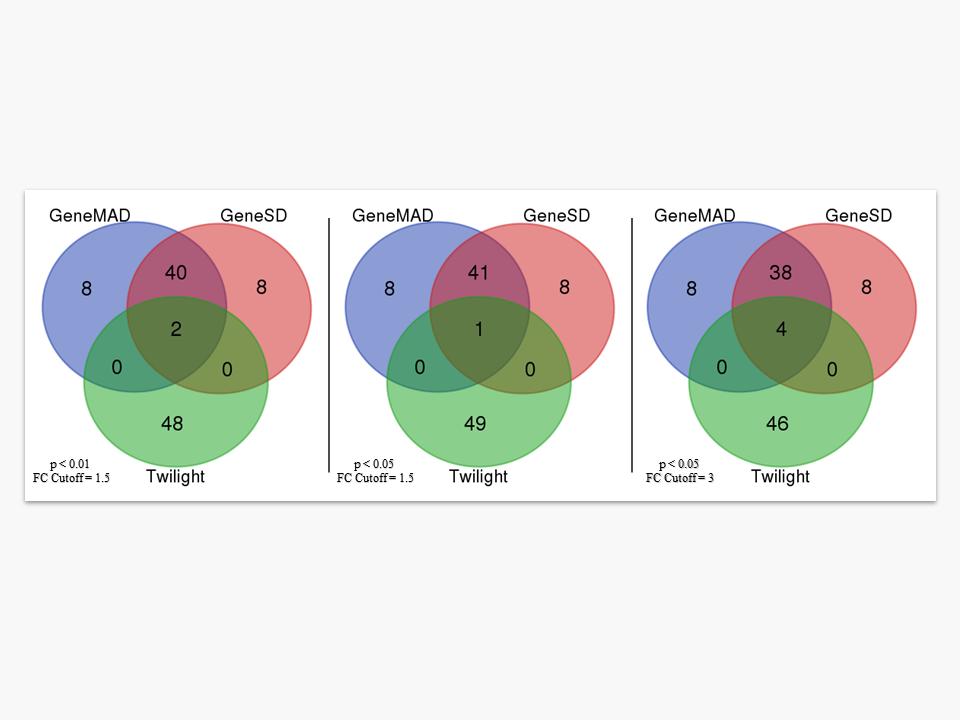


**Supplementary Figure 25.** Venn Diagram for top 50 genes for GSE49232


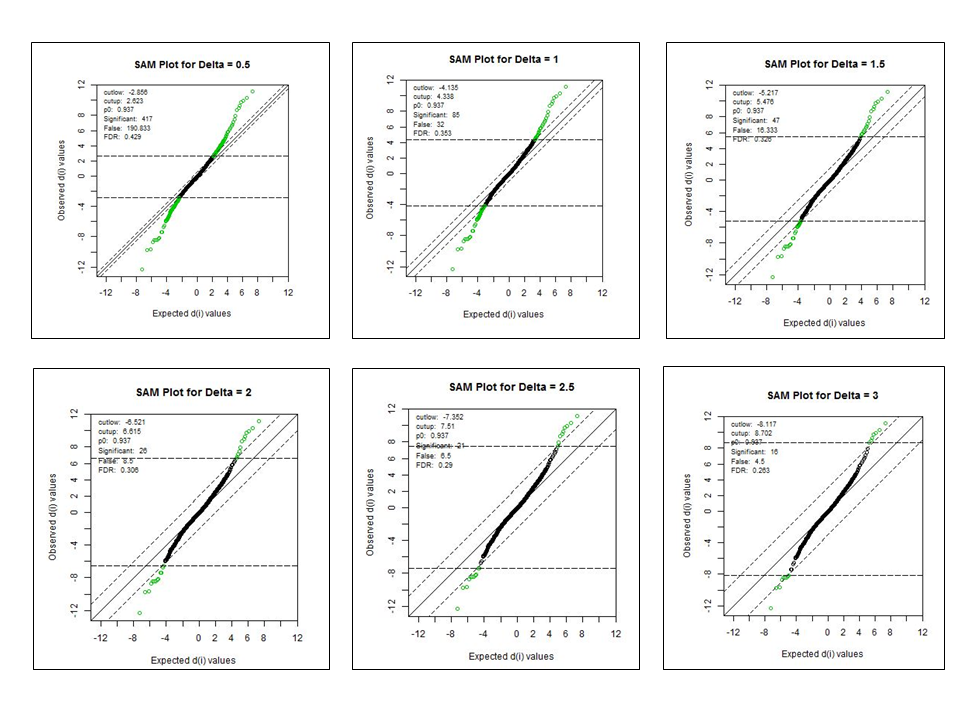


**Supplementary Figure 26.** SAM Analysis for GSE49232
